# Antimicrobial Action of Compounds from Marine Seaweed

**DOI:** 10.3390/md14030052

**Published:** 2016-03-09

**Authors:** María José Pérez, Elena Falqué, Herminia Domínguez

**Affiliations:** 1 mjperez@uvigo.es; 2 efalque@uvigo.es; 3

**Keywords:** seaweed, antimicrobial, *in vitro* and *in vivo* assays, extraction techniques

## Abstract

Seaweed produces metabolites aiding in the protection against different environmental stresses. These compounds show antiviral, antiprotozoal, antifungal, and antibacterial properties. Macroalgae can be cultured in high volumes and would represent an attractive source of potential compounds useful for unconventional drugs able to control new diseases or multiresistant strains of pathogenic microorganisms. The substances isolated from green, brown and red algae showing potent antimicrobial activity belong to polysaccharides, fatty acids, phlorotannins, pigments, lectins, alkaloids, terpenoids and halogenated compounds. This review presents the major compounds found in macroalga showing antimicrobial activities and their most promising applications.

## 1. Introduction

Marine organisms produce a variety of compounds with pharmacological activities, including anticancer, antimicrobial, antifungal, antiviral, antiinflammatory and others, and are potential sources of new therapeutic agents. Marine organisms survive and live within complex communities and in close association with others in a competitive and hostile environment. They produce complex secondary metabolites as a response to ecological pressure, such as competition for space, predation and tide variations. Some of these compounds are antimicrobials that inhibit or limit the development and growth of other competitive microorganisms.

Marine sessile organisms, such as algae, sponges and corals, have developed physiological adaptations, including the synthesis of bioactives which confer defense against grazers and/or the installation of epiphytes and fouling organisms [1,2,3]. Metabolites from green, brown and red marine algae may be useful for inhibiting bacteria, viruses, fungi and other epibionts (e.g., cytostatic, antiviral, antihelmintic, antibacterial, antifungal activity). Algae crude extracts and their fractionated or purified components also exhibit, for example, anticoagulant [4], antiviral [5], antioxidant [6], anticancer [7], and antiinflammatory [8] activities.

Microorganisms have developed new strategies to evade the action of antibiotics, leading to multiple drug-resistant bacterial strains. With increasing resistance of pathogens to antibiotics, there is a public health priority for exploring and developing cheaper and effective natural antimicrobial agents with better potential, less side effects than antibiotics, good bioavailability, and minimal toxicity [9]. It is also worthwhile to test the marine antimicrobials for possible synergism with existing drugs [10].

Updated information on this research area has been compiled recently, including the antiviral properties of marine organisms [11], the seaweed-associated bacterial and fungal communities [12], and the diversity and bioactives production of actinobacteria associated with the marine organisms [13]. Based on published studies, Vatsos and Rebours [14] reviewed the antimicrobial properties of seaweed extracts related with aquaculture. Eom *et al.* [15] reviewed the antimicrobial effects of phlorotannins from brown algae, in relation to the food and pharmaceutical industries. Abu-Ghannam and Rajauria [16] reviewed the algal antimicrobials with potential food applications. The studies showing biological activities of extracts from native and some non-native Brazilian seaweed [17] and the research progress concerning the isolation and structural elucidation of the secondary metabolites from the genus *Cystoseira* [18] were overviewed. The objective of this work is to gather the recently published information on the antimicrobial properties of compounds from seaweed, their extraction and the major applications.

## 2. Bioactive Compounds

In the food, pharmaceutical, cosmetic, cosmeceutical, nutraceutical and biomedicine industries, seaweed/macroalgae are used as a valuable source of bioactive compounds. Many compounds, being antiparasitic, antiviral or antibacterial, are effective [19]. The influence of some natural factors, such as the environmental conditions, including light, temperature or salinity, the life stage, reproductive state and age of the seaweed, and the geographical location and seasonality, allowed for the consideration that this antimicrobial activity was not attributed to a single compound, but it could be related to some of them and to a combination of metabolites.

Seaweed or macroalgae provide a great variety of metabolites and natural bioactive compounds with antimicrobial activity, such as polysaccharides, polyunsaturated fatty acids, phlorotannins and other phenolic compounds, and carotenoids.

### 2.1. Polysaccharides and Derived Oligosaccharides

The main components of green, brown and red seaweed are usually polysaccharides, which may have storage and structural functions. Cell walls of algae are composed of a variety of polysaccharides including alginic acid and alginates, carrageenans and agar, laminarans, fucoidans, ulvans and derivatives [20,21].

Their antimicrobial activity depends on some factors, such as their distribution, molecular weight, charge density, sulphate content (in sulphated polysaccharides), and structural and conformation aspects. In addition, oligosaccharides obtained by depolymerization of seaweed polysaccharides also induce protection against viral, fungal and bacterial infections in plants [22].

These polymeric carbohydrates structures are usually composed of various monosaccharides linked with different glucosidic bonds. Some algal polysaccharides, such as sulphated galactans of the red algae or ulvans of the green algae, have linear backbones containing dissacharide repeating units. Otherwise, alginic acids have linear molecules built up of different blocks of two monomerics units. Algal macromolecules include sulfated polysaccharides such as: carrageenan and agar from red algae; alginate, fucan and laminarinan from brown alga; and cellulose and ulvan from green algae.

#### 2.1.1. Alginates

**Figure 1:**
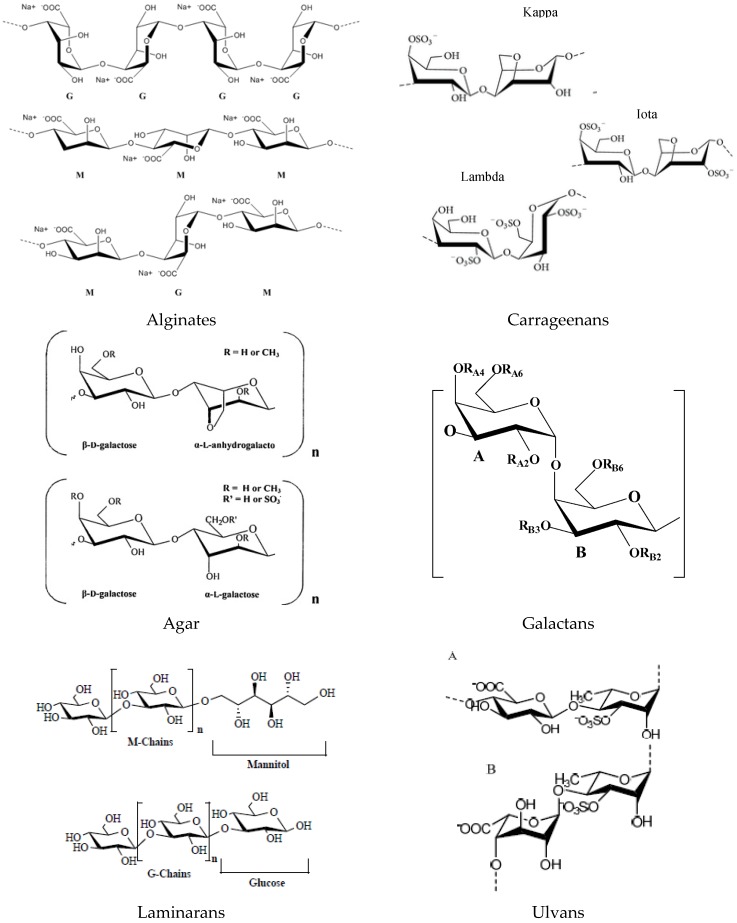
Basic chemical structures of mainly polysaccharides in macroalgae: alginates, carrageenans, agar, galactans, laminarans, fucoidans and ulvans.

Algins/alginates are available in both acid and salt forms. Alginic acids are linear copolymers of two uronic acids, β-d-mannuronic acid (M) and α-l-guluronic acid (G) linked in position 1→4. The salt forms (alginates), with several cations (Na^+^, K^+^, Mg^2+^ and Ca^2+^), are the major components of brown seaweed cell walls and also of the intracellular matrix [21]. Alginates are anionic polysaccharides and are made up of β-d-mannuronic acid (M) and α-l-guluronic acid (G), and alternating blocks of d-guluronic and d-mannuronic (M-M, G-G or M-G blocks) [22,23] (Figure 1). The molecular weight of alginate ranges generally between 500 and 1000 kDa.

#### 2.1.2. Carrageenans

Carrageenans are the major components of red seaweed cell walls, and this group of molecules is composed of linear polysaccharide chains with sulphate half-esters attached to the sugar unit. There are three general forms: kappa, lambda and iota, according to the degree of sulphation (Figure 1). Kappa carrageenan and iota carrageenan have one or two, respectively, sulphate groups per disaccharide unit and anhydrogalactose residue, and lambda carrageenan has three sulphate groups per disaccharide unit [22].

#### 2.1.3. Agar

Agar is a mixture of at least two polysaccharides, *i.e.*, agarose and agaropectin, extracted also from red seaweed with similar structural and functional properties as carrageenans. Agarose is the predominant fraction of agar, and it consists of high molecular weight polysaccharides composed of repeating units of (1→3)-β-d-galactopyranosyl-(1→4)-3,6-anhydro-α-l-galactopyranose (Figure 1). The structure of agaropectin, with a lower molecular weight than agarose, is essentially made up of alternating (1→3)-β-d-galactopyranose and of (1→4)-3,6-anhydro-α-l-galacto-pyranose residues.

#### 2.1.4. Galactans

Sulphated galactans are the main extracellular polysaccharides of red algae (but are also found in brown and green algae). Typical structure is a linear chain of galactoses; a chain of alternating 3-β-d-galactopyranose (G units) and 4-α-d-galactopyranose residues, or 4-3,6-anhydrogalactopyranose residues complete their structural backbone with presence of d-series (d unit) in carrageenans and l-series (l unit) in agarans. Other exceptional galactans present the dl-hybrids that enclose G unit attached to both d and l units [24].

#### 2.1.5. Laminarans

Laminaran is the principal storage polysaccharide of brown seaweed (e.g., *Laminaria* or *Saccharina* spp.) and their content can represent up to 32%–35% (d.w.). Laminarans are small glucans and are a linear polysaccharide composed of β-(1→3)-linked glucose, containing randomly β-(1→6) intra-chain branching, with a ratio around 3:1 [23]. The degree of polymerization varying between 20 to 50 units and the polymeric chains can be of two types according to their reducing end: M chains end with a d-mannitol residue, whereas G chains end with a glucose residue (Figure 1). The molecular weight is approximately 5000 Da depending on the degree of polymerization (usually 25) [25].

#### 2.1.6. Fucoidans/Fucans

Fucoidans and laminarans are considered as the main water-soluble polysaccharides of brown algae. Fucoidans are a complex and heterogeneous group of polysaccharides, which contribute to intercellular mucilage and are sulfated polysaccharides composed of l-fucose and sulphate ester groups with minor amounts of different molecules, which can vary from monosaccharides (*i.e.*, mannose, arabinose, glucose, galactose, xylose, *etc.*), acidic monosaccharides, acetyl groups to proteins [26].

Terms such as fucans, fucosans, fucose containing polymers or sulfated fucans have also been adopted for this group; however, finally, according to the IUPAC terminology, fucoidans is retained for polysaccharides of algal origin, and fucan sulphates (or fucans) to the similar polymers from marine invertebrates [21].

Various molecular weights, from 100 to 1600 kDa, have been reported in the literature for fucoidans [25] because they may differ considerably in their composition and chemical structure (degree of branching, substituents, sulphation and type of linkages).

Fucoidan composition varies with species and geographical origin, even within the same species. It appears that the prevalent core backbone structures are primarily the (1→3)-linked α-l-fucopyranosyl backbone structure, and secondarily the backbone structure composed of alternating α (1→3) and α (1→4)-linked l-fucopyranosyls [27]. The first group includes the fucoidans from *Laminaria* spp., *Analipus japonicus*, *Cladosiphon okamuranus*, and *Chorda filum* and the second group included fucoidans isolated from *Ascophyllum nodosum* and *Fucus* sp. However, sulphate- and acetyl-groups and some sugar residues may occur at C2 and/or at C4 positions.

#### 2.1.7. Ulvans

Ulvan designates a water-soluble sulphated polysaccharides extracted from the intercellular space and in the fibrillar wall of green seaweed (mainly *Ulva* sp.) and accounts from 18% to 29% of the algal dry weight [28]. These polysaccharides are mainly composed of glucuronic acid and iduronic acid units together with rhamnose and xylose sulfates, connected by α- and β-1→4 bonds, with an average molecular weight of ulvans ranging from 189 to 8200 kDa.

The main repeating disaccharide units reported are ulvanobiouronic acid 3-sulphate types containing either glucuronic or iduronic acid (Figure 1). Additionally, minor repeating units have been reported that contain sulfated xylose replacing the uronic acid or glucuronic acid as a branch on *O*-2 of the rhamnose-3-sulphate [29].

### 2.2. Lipids, Fatty Acids Ans Sterols

Algal lipids content in seaweed ranges from 0.12% to 6.73% (dry weight), and are composed mainly of phospholipids, glycolipids and non-polar glycerolipids (neutral lipids) [30]:

- Phospholipids are located in extra-chloroplast membranes and account for 10%–20% of total lipids in algae. They are characterized by higher contents of *n*-6 fatty acids, and the major fatty acids present are oleic, palmitic, stearic, arachidonic and eicosapentanoic acids. The most dominant phospholipid in algae is phosphatidylglycerol in green algae, phosphatidylcholine in red algae, and phosphatidylcholine and phosphatidylethanolamine in brown algae.
- Glycolipids are located in photosynthetic membranes and constitute more than half of the lipids in the main algal groups. They are characterized by high *n*-3 polyunsaturated fatty acids. Three major types of glycolipids are monogalactosyldiacylglycerides, digalactosyldiacylglycerides, and sulfoquinovosyldiacylglycerides [31].
- Triacylglicerol is the most prevalent neutral lipid, their content ranging from 1% to 97% with a function of storage and energy reservoir.

Fatty acids are carboxylic acids with aliphatic chains and prevalent even carbon numbers (C4-C28) that may be straight or branched, saturated or unsaturated. According to the double bond, fatty acids are classified as monounsaturated (MUFA) or polyunsaturated (PUFA) fatty acids, and the last one could be classified as *n*-3 or *n*-6 depending on the position of the first double bond from the methyl end. Green algae are rich in C18 PUFAs, mainly α-linolenic (C18:3 *n*-3), stearidonic (C18:4 *n*-3) and linoleic (C18:2 *n*-6) acids; red algae is rich in C20 PUFAs, mainly arachidonic (C20:4 *n*-6) and eicosapentaenoic (C20:5 *n*-3) acids, and brown algae exhibit both.

Oxylipins are the oxygenated products of fatty acids and are mainly derived from C16, C18, C20 and C22 PUFAs and confer innate immunity in response to biotic and abiotic stress, such as pathogenic bacteria and herbivores*.* [30].

**Figure 2:**
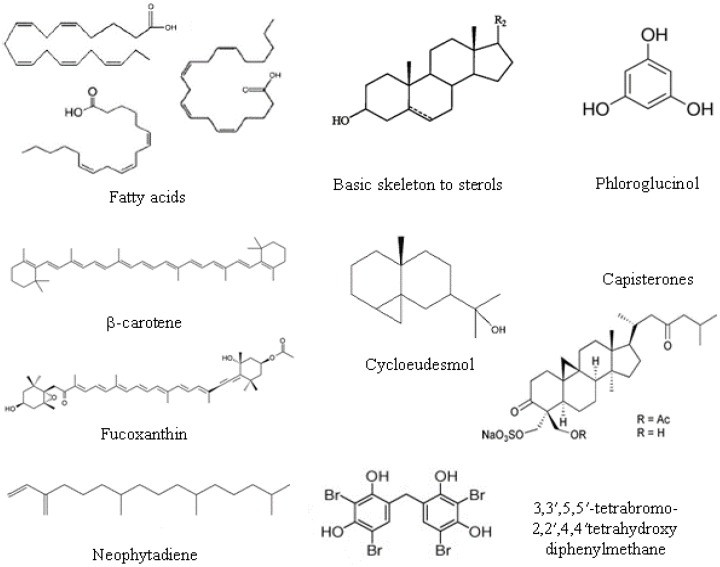
Chemical structures of fatty acids, sterol, phloroglucinol, carotenoids (β-carotene and fucoxanthin), terpenes (neophytadiene and cycloeudesmol) and a brominated compound.

Sterols are structural components of cell membrane and regulate membrane fluidity and permeability. They are composed of four rings (A–D) with a hydroxyl group in carbon-3, two methyl groups at C18 and C19 carbons and a side chain at C17 (see Figure 2). The main sterols in macroalgae are cholesterol, fucosterol, isofucosterol, clionasterol [30].

### 2.3. Phenolic Compounds

Phenolic compounds are secondary metabolites because they are not directly involved in primary processes such as photosynthesis, cell division or reproduction of algae. They are characterized by an aromatic ring with one or more hydroxyl groups and the antimicrobial action is due to the alteration of microbial cell permeability and the loss of internal macromolecules or by the interference with the membrane function and loss of cellular integrity and eventual cell death [16].

Chemically, structures ranging from simple phenolic molecules to complex polymers with a wide range of molecular sizes (126–650 kDa) have been described [26]. Polyphenols could be divided into phloroglucinols and phlorotannins. Phloroglucinol contains an aromatic phenyl ring with three hydroxyl groups (Figure 2). Phlorotannins are oligomers or polymers of phloroglucinol with additional halogen or hydroxyl groups; and, according to the inter-linkage, phlorotannins can be subdivided into six specific groups: (i) phlorethols (with aryl-ether linkage); (ii) fucols (with aryl-aryl bonds); (iii) fucophlorethols (with ether or phenyl linkage); (iv) eckols (with dibenzo[1,4]dioxin linkages); the less frequent (v) fuhalols (with *ortho-*/*para*-arranged ether bridges containing an additional hydroxyl on one unit); and (vi) carmalols (with dibenzodioxin moiety) [32]. The presence of simple phenols, such as hydroxycinnamic and benzoic acids and derivates, and flavonoids were reported in the green seaweed [33], but brown seaweed has higher contents of phenolic compounds than green and red macroalgae. The typical phlorotannin profile from brown algal with antimicrobial activity mainly consists of phloroglucinol, eckol and dieckol [19].

### 2.4. Pigments

Algae as photosynthetic organisms can synthezise the three basic classes of pigments found in marine algae:chlorophylls, carotenoids and phycobiliproteins, allowing classification of seaweed into Chlorophyceae (green algae), Phaeophyceae (brown algae) and Rhodophyceae (red algae). The green color is due to the presence of chlorophylls *a* and *b*, the greenish brown color is attributed to the fucoxanthin, chlorophylls *a* and *c*, and responsible for the red color are the phycobilins, such as phycoerythrin and phycocyanin [34].

The antimicrobial mechanism proposed for carotenoids could lead to the accumulation of lysozime, an immune enzyme that digests bacterial cell walls [16]. Carotenoids are present in all algae and are lipid-soluble, natural pigments composed of eight units of five carbons, namely tetraterpenoids, with up to 15 conjugated double bonds. Carotenoids are usually divided in two classes: carotenes (when the chain end with a cyclic group, containing only carbon and hydrogen atoms) and xantophylls or oxycarotenoids (which have at least one oxygen atom as a hydroxyl group, as an oxy-group or as a combination of both). β-Carotene is the most common carotene (Figure 2), whereas lutein, fucoxanthin (Figure 2) and violaxanthin belong to the xanthophylls class [35]. β-Carotene, lutein, violaxanthin, neoxanthin and zeaxanthin are found in green seaweed species; α- and β-carotene, lutein and zeaxanthin are present in red seaweed, and β-carotene, violaxanthin and fucoxanthin are found in brown algae [34].

### 2.5. Other Compounds

Seaweed is also able to produce other secondary metabolites with a broad range of antifungal, antiviral, antibacterial, antimacrofouling and antiprotozoan activities, such as terpenes, alkaloids, lectins or halogenated compounds.

#### 2.5.1. Lectins

Lectins are natural bioactive ubiquitous proteins or glycoproteins of non-immune response that bind reversibly to glycans of glycoproteins, glycolipids and polysaccharides possessing at least one non-catalytic domain causing agglutination.

Algal lectins differ from terrestrial lectins because they are monomeric, low molecular weight proteins, exhibiting high content of acidic amino acids, with isoelectric point in the range of 4–6. They do not require metal ions for their biological activities, and most of them show higher specificity for oligosaccharides and/or glycoproteins than for monosaccharides. Based on the binding properties to glycoproteins, algal lectins are categorized into three major categories: complex type N-glycan specific lectins, high mannose (HM) type N-glycan specific lectins and lectins with specificity to both the above types of N-glycans [36]. Lectins from marine organisms are also classified into C-type lectins, F-type lectins, galectins, intelectins, and rhamnose-binding lectins [37].

#### 2.5.2. Alkaloids

An alkaloid is a compound that has nitrogen atom(s) in a cyclic ring. Numerous biological amines and halogenated cyclic nitrogen-containing substances are included in the term alkaloid. The latter is specific to marine organisms and marine algae. They could not be found in terrestrial plants. Alkaloids in marine algae were classified in three groups as follows [38]: (i) phenylethylamine alkaloids; (ii) indole and halogenated indole alkaloids; and (iii) other alkaloids, such as 2,7-naphthyridine derivatives. Alkaloids isolated from marine algae mostly belong to 2-phenylethylamine and indole groups. Halogenated alkaloids are specific for algae, being bromine- and chloride-containing alkaloids particularly dominant in Chlorophyta. Most of the alkaloids of the indole group are concentrated in Rhodophyta [39].

#### 2.5.3. Terpenes

Terpenes represent one of the major classes of metabolites produced by marine algae. Chemically, they are derived from the five-carbon precursor isopentenyl pyrophosphate, and are classified into hemiterpenes (C5), monoterpenes (C10), sesquiterpenes (C15), diterpenes (C20), sesterterpenes (C25), triterpenes (C30) and polyterpenes (>C30). Chlorophyceae contain cyclic and linear sesqui-, di-, and triterpenes, while Rhodophyceae are characterized by a high structural diversity of halogenated secondary metabolites whose polyhalogenated monoterpenes exhibit a wide range of antimicrobial activities [40].

Some terpenes were isolated from seaweed [41], such as the diterpene neophytadiene (Figure 2); the sesquiterpenes cartilagineol, obtusol and elatol; the diterpene aldehyde halitunal which shows antiviral activity; the dolastane diterpenes 4-hydroxy-9,14-dihydroxydolasta-1(15),7-diene (**1**) and 4,7,14-trihydroxydolasta-1(15),8-diene (**2**); the diterpenoid halimediatrial and halimedalactone; the antibiotic cyclopropane containing sesquiterpene cycloeudesmol; the sesquiterpenoid puupehenone and its derivatives; *etc*. Many of them present antiviral activity; others, such as the sesquiterpene (−)-elatol are antifouling agents, and capisterones, which are triterpene sulphate esters (Figure 2), exhibit potent antifungal action.

#### 2.5.4. Halogenated Compounds

Other compounds found in macroalgae are halogenated metabolites, mainly brominated [1,33]: furanones 4-bromo-3-butyl-5-(dibromoethylene)-2(5*H*)-furanone and 4-bromo-5-(bromomethylene)-3-butyl-2(5*H*)-furanone; bromoditerpenes, such as 12 S-hydroxybromospha-erodiol, bromosphaerone and isoparguerol and their derivatives; bromophenols, such as bis(2,3-dibromo-4,5-dihydroxybenzyl) ether, 5-bromo-3,4-dihydroxybenzaldehyde and 3,3′,5,5′-tetrabromo-2,2′,4,4′tetrahydroxydiphenylmethane (Figure 2); and polar compounds, such as 2,3-dibromo-4,5-dihydroxyphenyl-ethylamine and 3,4-dihydroxyphenyl-ethylamine.

## 3. Assesment of Antimicrobial Activity

Several methods are widely used by researchers to detect and to measure the antimicrobial activity of algal extracts or their metabolites. Authors refer most often to *in vitro* and sometimes *in vivo* assays, but different algal extracts quantities and microorganisms are tested, making it difficult to unify results. In some cases, an initial *in vitro* screening is followed by an *in vivo* study, but most studies on the antimicrobial effects of seaweed are either only *in vitro* or only *in vivo*. Vatsos and Rebours [14] report the different assays published about antimicrobial activity of seaweed extracts in aquaculture. Here, we will exhibit the *in vitro* and *in vivo* methods more commonly referred to by authors in different fields.

### 3.1. In Vitro Assays

#### 3.1.1. Diffusion Agar Tests

The method widely used by authors to evaluate antibacterial and antifungical activity is the agar disk diffusion test. Briefly, it is based on the use of sterile paper disks impregnated with the algal extract to be evaluated (concentrations varies greatly from one author to another [42,43,44,45,46,47]) and are deposited in an agar plate with a particular bacterial or fungal culture (the suspensions of microorganisms ranging between 10^5^ cells/mL [48] and 10^8^ colony forming units (cfu)/mL [42,49]. Soluble extracts diffuse into the culture medium (generally Mueller-Hinton Agar used for bacteria and Saboureaud Agar plates, with or without chloramphenicol for fungal strains) and after the incubation time suitable for each microorganism, and the halos of inhibition of microbial growth around the disks are recorded and measured. Bansemir *et al.* [47] refer to the use of *p*-iodo nitrotetrazolium violet, 5% in 50% aqueous ethanol to detect the inhibition zone because living bacteria produce a red-colored compound by reaction with the coloring solution and the inhibition zone appears colorless.

The diffusion agar test can be performed also preparing wells in agar. Thanigaivel *et al.* [50,51] prepared Petri plates containing the culture medium and inoculated them by swabbing the microorganism chosen. Once dried under sterile conditions, wells 6–8 mm in diameter were made with a punch, and the algal extracts were placed into them. Shanmughapriya *et al.* [52] performed the agar well diffusion method using porcelain beans. The plates were incubated under the optimal conditions for each microorganism, and the presence or not of an inhibition zone was determined.

#### 3.1.2. Growth Inhibition Assay

García Bueno *et al.* [53] tested the antibacterial activity of water-soluble seaweed extracts in 96-well plates. Bacterial growth in the presence of algal extracts was monitored by measuring optical density (OD) at 490 nm every 30 min for 24 h. After incubation, the intensity of growth in the presence of the tested compounds and controls was compared. Cox *et al.* [54] assessed the antibacterial activity of varying concentrations of a hydrophilic extract from *Himanthalia elongata* in carbohydrate and protein model food systems, and Dubber and Harder [55] used a highly sensitive growth inhibition assay that recorded the fluorescence intensity of stained DNA.

Pinteus *et al.* [43] determined the antifungal activity by following the yeast growth in yeast extract peptone dextrose (YPD) broth in the presence or absence of extracts. The growth was monitored spectrophotometrically (OD 600 nm) after 20 h of incubation. The growth inhibition was assayed as the percentage of change in turbidity in the presence or absence of algal extracts.

#### 3.1.3. Minimun Inhibitory Concentration (MIC) Determination

MIC represents the lowest concentration of crude or purified algal extracts that inhibits the bacterial or fungal growth. The concentration serial broth (micro) dilution assay has been used in several studies [56,57,58].

Boisvert *et al.* [56] used a microdilution method using 96-well flat bottom microplate. Two-fold serial dilutions of extracts samples were made by repeated transfers of 125-μL volumes of tryptic soy broth (TSB) in a 96-well flat-bottom microplate. Each well was inoculated with 50 μL of bacterial suspension adjusted to 10^5^ cfu/mL. Optical density was monitored every 10 min with a microplate spectrophotometer, and percentage of inhibition was calculated. Bazes *et al.* [57] assayed different concentrations of algal extracts incubated with 2 × 10^8^ cells/mL for 48 h at 20 °C; after, that growth was monitored by measuring OD600.

Hellio *et al.* [58], determined the MIC of crude algal extracts via the macrodilution method. They assayed the extract concentration between 96 and 4 μg/mL, and 2 × 10^8^ cfu/mL were placed in a liquid medium suitable for microorganisms tested with algal extracts.

The MTT (methylthiazol tetrazolium) cell proliferation assay was used by Spavieri *et al.* [59] to evaluate the antitubercular activity against *Mycobacterium tuberculosis.* MICs values were measured using aproppiated bacterial media in a 96-well flat bottom plate. Serial extract dilutions (final concentrations of 1–256 μg/mL) and 100 μL with 2.5 × 10^7^ *Mycobacterium tuberculosis* strain H37Rv were added to the wells. The plate was incubated with gentle rocking for 7 days at 37 °C. Ten μL of MTT were added to each well, and the plates were then incubated for a further 24 h. MICs were recorded as the lowest concentration at which a purple precipitate of formazin did not appear in the wells.

#### 3.1.4. Other Studies

The antiprotoozal activity of 90 to 123 µg/mL of brown algae extracts against 10^4^/50 µL bloodstream forms of *Tripanosoma brucei rhodesiense*, 5000/100 µL trypomastigote forms of *T. cruzi*, and 10^5^ amastigotes of *Leishmania donovani* were measured by Spavieri *et al.* [59] using 96-well microtiter plates; after incubation, microplates were read and data analyzed using a microplate reader.

Bazes *et al.* [57] assayed inhibition of phytoplankton growth by algal extracts. The effect of different concentrations of algal extracts on phytoplanctonic strains was assessed after 72 h by estimating the chlorophylla-*a* content. The percentage of growth inhibition was calculated. Additionally, they determined the percentage of inhibition of the germination of spores of *Ulva* sp. (600/mL) in plastic Petri dishes after incubation for 5 days at 20 °C under 24 h light.

Bouhlal *et al.* [60] determined the antiviral activity against Herpes simplex virus tipo1of algal extracts. Vero cells (African green monkey kidney cell line) were infected with HSV-1 suspension without or in the presence of different dilutions of the extracts. After incubation, antiviral activity was evaluated by the neutral red dye method. The 50% effective antiviral extract concentration (EC_50_) was expressed as the concentration that achieved a protection of 50% of virus-infected cells. OD measured at 540 nm was directly related to the percentage of viable cells, which was inversely related to the cytopathic effect.

Wang *et al.* [61] reviewed most of the studies on antiviral effects of marine polysaccharides carried out both *in vitro* or in mouse model systems. Therefore, further studies are needed in order to investigate their antiviral activities in human subjects.

### 3.2. In Vivo Assays

In the literature related to the assessment of antimicrobial activity of the crude extracts or fractions of algae, the *in vivo* assays are less numerous. *In vivo* assays depend critically on the target organism or substrate varying between one another. Vatsos and Rebours [14] refer to farmacodynamic, pharmacokinetic studies and artificial challenges like survival rate, progress of disease and severity of signs or lessions in the studies applied to aquaculture. For possible use in humans, they are much more restricted, so the detection assays *in vitro* antimicrobial activity will be fundamental. In this section, some of the *in vivo* assays based on published studies are collected.

Manilal *et al.* [62] assayed *in vivo* the therapeutic potential of crude algal extracts, which were rationalized with commercial shrimp feed and orally administered for different durations, followed by the artificial bacterial challenge experiment. The determination of the effective dose of medicated feed is necessary to perform the *in vivo* assays; after three weeks of feeding trial, ten shrimps from each group (medicated and control groups) were individually challenged with four species of live *Vibrio*. The challenged animals were monitored for a period of two weeks for mortality and infections. During this period, the shrimps were fed continuously with their respective feeds. The cause of death/infection was ascertained by reisolating the respective organism from the shrimp body and subjecting the isolates to standard biochemical tests. The morbidity and mortality were recorded daily and final mortality was evaluated after two weeks of postchallenge, and percentage of mortality was calculated.

Thanigaivel *et al.* [9,50,51] performed assays to demonstrate the antibacterial protection for fish by inmersion method. Therefore, for the experimentally induced infection of fish, different concentrations (10^1^ to 10^5^) of pathogenic bacterial cell suspension were added to the water where healthy fish were maintained. For treatment different concentrations varying from 50 mg/L to 500 mg/L of prepared seaweed extract were also added. Syntomatic or dead fish were recorded.

## 4. Production of Antimicrobials from Seaweed

Due to the different chemical natures of potential antimicrobials, the conditions leading to their maximal production, extraction and recovery should be optimized for each particular case. This section summarizes the information for the production of compounds with antimicrobial action, including an overview of the biotic and abiotic factors influencing their content in seaweed and the effect of processing stages. Most studies refer to crude extracts, with different components that may act separately or synergistically to exert antimicrobial activity.

### 4.1. Factors Affecting the Content of Antimicrobials in Seaweed

The chemical composition of the algae and the antimicrobial activities vary with species, physiological status, the region of the thallus, environmental aspects (climate, location, salinity, temperature), pollution, growth conditions, collection time and epiphytic organisms [63,64,65].

Different studies confirmed the variation in chemical compositions and the antimicrobial action according to season. Most authors detected maximal antimicrobial potential in spring, probably due to the predominance of some active compounds in this period [53,66,67,68]. However, a different variation of the phenolic composition and antibacterial/antioxidant activities was observed, with the highest inhibitory activity in spring and summer, and phenolic content and antioxidant activity in late winter [64]. Other studies have shown a lack of activity during some seasons [68], no clear seasonal variation of antifungal [66] and antibacterial activities [55], or a different pattern among algal classes, whereas Phaeophyceae showed an absence of activity in certain seasons, Rhodophyceae showed variations along seasons and Chlorophyceae were active throughout the year [69]. The effect of the latitude and related environmental factors on the phenolic content and phlorethol type in *Sargassum muticum* collected in different European coasts was reported [70].

Seaweed-associated microbial communities are highly diverse and rich sources of exceptional molecular structures [71]. Over a long evolutionary period, the marine organisms sharing a common environment have established associations [13]. Bacteria present either outside or inside the algal cells protect the host against pathogens by producing bioactive compounds. Despite epiphytic microorganisms showing high antimicrobial activity [71,72,73], they are not the object of this work. In most mentioned studies, epiphytic bacteria have been removed by washing.

### 4.2. Extraction of Antimicrobials from Seaweed

The bioactives yield can be affected by the method of conditioning and extraction. The drying stage is important, since loss of volatile antimicrobials present in fresh algae (hydrogen peroxide, terpenoid and bromo-ether compounds and volatile fatty-acids) could occur at high temperatures [74]. These authors observed increased permeability of cell membranes and reported that extracts from dried algae processed at high temperatures presented wider inhibition zones for *Salmonella enteritidis*, *Pseudomonas aeruginosa*, *Listeria innocua*, and on both clinical and food isolates of *Staphylococcus aureus*. Similarly, Chambers *et al.* [75] reported that crude ethanol extracts of dried *Chondrus crispus* had a lower MIC against the growth of bacteria and phytoplankton species than the extracts from the fresh seaweed. Cox *et al.* [76] reported a reduction of the total phenolic content and antioxidants from *H. elongata* during hydrothermal processing and drying, respectively. Drying followed by boiling increased the phytochemicals content and enhanced the inhibitory potential.

The fermentation of an *Eisenia bicyclis* broth with *Candida utilis* YM-1 during one day enhanced the phenolic content (eckol, dieckol, dioxinodehydroeckol, and phlorofucofuroeckol-A), and the antimicrobial activity against methicillin-resistant *S. aureus* and food-borne pathogenic bacteria [77].

Cell wall and storage polysaccharides from green, brown and red seaweed, including ulvans, alginates, fucans, laminarin and carrageenans can trigger defense responses in plants against pathogens. The oligosaccharides obtained by depolymerization of seaweed polysaccharides also induce protection against viral, fungal and bacterial infections in plants, leading to the accumulation of proteins and compounds with antimicrobial activities [22].

Choi *et al.* [67] reported that irradiation with Co-60 gamma rays at 1–30 kGy increased the Hunter color *L* value of *Ecklonia cava* ethanol extracts in a dose-dependent manner, without altering the antimicrobial activity, but the *a* and *b* values and the antioxidant properties decreased with irradiation. An acute toxicity test using mice and a cytotoxicity assay on the murine macrophage cell line RAW264.7 suggested that irradiated *E. cava* extracts can be safely used by humans at moderate doses.

#### 4.2.1. Solvent Extraction

Various methods have been proposed to extract the bioactive compounds from seaweed, using organic solvents. The yield of extractables and antimicrobials from the different species of seaweed is solvent dependent. Systematical evaluation and optimization of the solvent is necessary for accurate and reproducible preparation of extracts. Several studies aimed at selecting the best solvent, which was usually one of the following: water, methanol, ethanol, acetone, ethyl acetate, dichloromethane, chloroform, diethyl ether and hexane [58,68,78,79,80,81]. Different authors have compared the different families of seaweed regarding antibacterial activity [6,69,82,83,84]. Table 1, Table 2 and Table 3 present some examples of the microorganisms inhibited by solvent extracts from red, green and brown seaweed, respectively.

In most cases, the extracts produced using organic solvents appear more efficient [14,79], probably because the inhibition mechanisms are due in part to the hydrophobic nature of some components, such as fatty products [3,106]. Other works confirmed that polar extracts have higher antibacterial activity [98,113,114,115]. Abundant studies have confirmed that alcoholic solutions and/or hydrophilic solvent mixtures provided better activity, *i.e.*, methanol and acetone extracts were more active than those in lipophilic solvents as chloroform/methanol [68,74]. Krish and Das [68] reported that the ethanol and methanol extracts from *Cladophora rupestris* were more active than the ethyl acetate ones, but all of them were less potent than ampicillin. Methanol was found to be more effective in a number of studies [55,66,84,98,113,116,117], but also ethanol [80], acetone [78,82,101], water [98], ethyl ether [3] and ethyl acetate [74,118] were tried. Rajauria *et al.* [107] found considerable variations in the extraction yield and antimicrobial activity among different concentrations of methanol, 60% methanol providing optimal performance from *H. elongata*. However, the optimal solvent depends on many factors, particularly on the target solutes and microorganisms, and a general recommendation is not possible, *i.e.*, Cox *et al.* [6] selected methanol for brown and acetone for red and green seaweed species, whereas Shanmugam *et al.* [119] selected methanol for red and for brown seaweed, and acetone was better for green species.

The intensification of the extraction stage can reduce the time and energy demand. Patra *et al.* [120] proposed microwave-assisted hydrodistillation to extract the essential oil from *Enteromorpha linza*. The major active compounds, such as *n*-hexanal, 2-chloro-5-methyl-4-(2-thienyl) pyrimidine, tetradecanoic acid, pentadecanoic acid, (*z*,*z*)-6,9-*cis*-3,4-epoxy-nonadecadiene, 13-octadecenal and azetidine, individually or synergistically inhibited the growth of pathogenic bacteria. Kadam *et al.* [121] reported the beneficial effect of ultrasound assisted extraction on the laminarin yields from *Ascophyllum nodosum* and *Laminarina hyperborea*. The purified extracts have shown inhibition of *Staphylococcus aureus*, *Listeria monocytogenes*, *E.coli* and *Salmonella typhimurium.*

#### 4.2.2. Alternative Solvents

In addition to conventional solvents, alternative green technologies have been proposed for the extraction of algal components [122]. Pressurized solvents, *i.e.*, supercritical carbon dioxide (SC-CO_2_) or subcritical water extraction, are considered green, environmentally friendly methods. Plaza *et al.* [106] proposed the use of pressurized solvent extraction of *H. elongata* with hexane, ethanol and water and did not observe that increased temperatures significantly chaged the antimicrobial activity of the extracts, mainly consisting of fatty acids (palmitoleic and oleic acids), sterols, alkanes and phenols. Boisvert *et al.* [56] used ethanol under pressurized liquid conditions to extract antimicrobials form brown and green algae.

Sivagnanam *et al.* [112] proposed the supercritical (SC)-CO_2_ (45 °C, 250 bar) extraction using ethanol as a co-solvent to produce extracts with phenolic content and antioxidant properties higher than those with conventional solvents from brown seaweed (*Saccharina japonica* and *Sargassum horneri*). However, the acetone-methanol extracts exhibited better antimicrobial activities.

Meillisa *et al.* [111] proposed subcritical water hydrolysis (200–280 °C, 1.3–6.0 MPa) to extract antibacterial compounds from *S. japonica*, previously de-oiled by SC-CO_2_ extraction. Strong antibacterial activity against two Gram-negative (*E. coli* and *S. typhimurium*) and two Gram-positive bacteria (*S. aureu*s and *B. cereus*) was found using acetic acid to aid in the hydrolysis. The MIC values ranged from 1.60 to 3.50 mg/mL, and the optimal hydrolysis temperatures were 240 °C against *B. cereus* and *S. typhimurium* and 280 °C against *E. coli* and *S. aureus*. Since acetic acid had antibacterial activity at 1%, and only 0.48%–0.6% acetic acid remained in the hydrolysate, the addition of acetic acid in the hydrolysis process can improve the extraction of antibacterial substances from the alga.

#### 4.2.3. Sequential Extraction and Purification

A series of extraction stages has been proposed either from the algal material or as a purification strategy of crude extracts. Examples of the first alternative include a sequential extraction of *H. elongata* with a mixture of low polarity solvents (chloroform, diethyl ether and *n*-hexane) followed by the extraction of the residue with 60% aqueous methanol and the pooled crude extract was further partitioned using water and ethyl acetate [54]. Amorim *et al.* [90] repeated hot aqueous extraction on the raffinate to obtain a crude sulfated polysaccharide from *G. ornate*.

Crude extracts have been purified or refined in bioactive-guided fractionation processes, including sequential solvent extraction and/or chromatography. Águila-Ramírez *et al.* [3] purified crude acetone/methanol extracts with ethyl ether and with butanol. Jaswir *et al.* [81] fractionated a methanolic extract of *Sargassum plagyophillum* using solvents of increasing polarity. Kim *et al.* [123] fractionated an 80% ethanol crude extract of *S. muticum* with *n*-hexane, dichloromethane, ethylacetate, and butanol.

De Felício *et al.* [124] partitioned *Bostrychia tenella* extracts using *n*-hexane and dichloromethane and further choromatographic fractionation. Park *et al.* [102] reported the fractionation of the 4:1 methanol:water extract from *E. linza*, using chloroform, Sephadex LH-20 gels and reverse-phase HPLC (high-performance liquid chromatography) using a C18 column to yield pure compounds. Ismail *et al.* [125] fractionationated aqueous extracts from *Zonaria tournefortii* by flash chromatography in a C18 column. Kavita *et al.* [83] fractionated a *L. papillosa* extract using silica gel chromatography with a solvent mixture of increasing polarity. Rodrigues *et al.* [96] reported the fractionation of dichloromethane extracts of *Sphaerococcus coronopifolius*, using eluents of increasing polarities in normal phase vacuum liquid chromatography on silica. Bromoditerpenes were the active compounds in fractions showing growth inhibition against *S. aureus* and *C. albicans*.

## 5. Applications

### 5.1. Biofouling

All surfaces immersed in the marine environment are susceptible to being colonized by biofouling communities, becoming one of the biggest problems faced by the shipping companies. Since marine seaweed thalli contain less epibionts compared to biofilms on inanimate substrata, probably because they produce secondary metabolites which prevent bacterial attachment and growth. Several species of seaweed exhibit different patterns of production of bioactive compounds depending on the seasonal variation of the fouling pressure, and a higher production in spring and summer was reported, corresponding to the algae and invertebrates spawning season, and maximal values for water temperature and light intensity [2,3]. In the past, paints were formulated with toxic compounds such as As, Hg or TBT (tributyltin). Consciousness of the marine pollution, and its effects on both the food-chain and the genetic mutations of exposed animals, led to increased restrictions on the use of toxic biocides in the manufacture of antifouling products. Alternative solutions based on antifoulants with broad-spectrum activity and low toxicity to non-target organisms, stability in a paint formulation, and commercial availability are sought [3,75]. Marine macroalgae could be an interesting source, since fatty acids, lipopeptides, amides, alkaloids, terpenoids, lactones, pyrroles and steroids could be active antifoulants [31]. Barreto and Meyer [126] reported the isolation of lanosol ethyl ether from *Osmundaria serrata* and its antimicrobial activity against various terrestrial bacteria and fungi, and marine bacteria isolated from the surface of the seaweed. Vairappan *et al.* [127] isolated brominated sesquiterpenes, acethylmajapolenes, halogenated sesquiterpenes, some halogenated acetogenins and bromoalenes from *Laurencia* sp. These compounds displayed antibacterial activity against some marine bacterial strains. Sesquiterpene-type compounds could be the active compounds in ethyl ether extract of *Laurencia johnstonii*, which showed inhibition zones comparable to those obtained with erythromycin [3].

The antifouling activity can be evaluated against bacteria, fungi, diatoms, macroalgal spores, mussel phenoloxidase activity, and barnacles and testing under real world conditions to predict performance, *i.e.*, in static docks, is recommended. The seasonal and geographical variations of shipping paths should be considered for the formulation of antifoulants effective against a broad spectrum of organisms and marine environments.

Hellio *et al.* [58] found high levels of *in vitro* antifouling activity of aqueous, ethanolic and dichloromethane extracts from 30 marine algae from Brittany coast (France) against 35 isolates of marine bacteria. About 20% of the extracts were found to be active and non-toxic to oyster and sea urchin larvae and mouse fibroblasts. Hellio *et al.* [2] reported that dichloromethane extracts of Rhodophyceae were the most active among antifouling agents against different macroalgal species from the Bay of Concarneau (France).

Águila-Ramírez *et al.* [3] tested six seaweed extracts against the growth of selected species of marine colonisers (bacteria, fungi and microalgae). The ether extracts of *Laurencia johnstonii*, *Ulva lactuca* and *Dictyota flabellata* were more active in comparison with buthanol extracts against *S. aureus*. The most potent against the marine microalgae *Rhodosorus magnei*, *Neorhodella cyanea* and *Prymnesium calathiferum* were *U. lactuca* and *L. johnstonii* (MIC: 0.1–1 µg/mL) and *C. fragile* (MIC: 1–10 µg/mL).

Ben Redjem *et al.* [128] compared the organic extracts of eleven species of brown macroalgae collected from northern coasts of Tunisia and reported the highest antibacterial activity of the Cutleriales and Dictyotales, and the best inhibition against the diatom *Chaetoceros calcarans* for *Scytosiphonales*. Mathan *et al.* [129] reported that acetone/methanol extracts further fractionated with ethyl ether; with butanol, they were active against *Halomonas marina*, *Polaribacter irgensii*, *Pseudoalteromonas elyakovii*, *Rosevarius tolerans*, *Vibrio aestuarianus*, *V. anguillarum* and *V. pomeroyri*.

Some studies confirmed the lack of toxicity of algal extracts. Bazes *et al.* [57] observed antifouling activity of extracts from *Ceramium botryocarpum* on well plates against bacteria associated with immersed surfaces: *Vibrio* sp. and *Pseudovibrio denitrificans*. Chambers *et al.* [75] evaluated the antifoulant potential of *Chondrus crispus* on marine bacterial, phytoplankton and macroalgae strains. The extract from dried seaweed was more active than the fresh extract, and the longer shelf life of a dry product might be preferable. Macroalgae tests confirmed the anti-germination action against *U. pinnatifida* and *U. intestinalis* spores and a test panel sea exposure at docks, and confirmed its activity for six weeks and better performance than the same paint containing chlorothalonil. However, in open sea trials the extract showed less fouling coverage than chlorothalonil.

### 5.2. Aquaculture

Diseases of microbial origin are responsible for high mortality rates and lesions on fish skin, with important economic losses in aquaculture. According to the review by Vatsos and Rebours [14], in the last 20 years, there has been an increasing interest in various seaweed extracts as prophylactic and/or therapeutic agents, namely, red and brown seaweed, those exhibiting significant antimicrobial properties against fish and shrimp.

Vibriosis is a common disease affecting warm and cold water fish and seafood species caused by bacteria of the genus *Vibrio*, such as *Vibrio anguillarum*, *V. ordalii*, *V. harveyi*, *V. vulnificus*, *V. parahaemolyticus*, *V. alginolyticus*, *V. salmonicida*, which can accumulate in the fish flesh. Treatment with commercial antibiotics can cause toxicity to the animals and the release of chemical residues into the environment. Some *Vibrio* strains, including *V. harveyi*, *V. parahaemolyticus* and *V. splendidus*, are resistant to several antibiotics. Cavallo *et al.* [45] observed different susceptibilities of the chloroform/methanol extracts of red and green algae on *Vibrio* species using the disc diffusion method. *G. longissima* offered the broadest antibacterial spectrum, showing activity against *V. ordalii*, *V. salmonicida*, *V. alginolyticus* and *V. vulnificus*, followed by *Cladophora rupestris* extract, active against three species, and *Chaetomorpha linum* and *G. dura* extracts against two *species*, but none of the extracts tested demonstrated inhibition against *V. splendidus* and *V. harveyi.*

Mendes *et al.* [74] characterized the antimicrobial activity of solvent extracts from *Gracilaria vermiculophylla*, *Porphyra dioica* and *C. crispus*, both from wild and from integrated multi-trophic aquaculture. The higher potency in extracts from aquaculture species, when compared with the wild ones may be due to the environmental conditions, such as the presence of larger concentration of compounds from the breeding tanks, the constant water motion and aeration, and to the exposure to higher light intensities during longer periods of time. Aquaculture extracts of *G. vermiculophylla* and *P. dioica* presented a higher content of fatty acids. The ethyl acetate extracts predominated saturated fatty acids, especially palmitic acid, followed by polyunsaturated and monounsaturated fatty acids.

Bansemir *et al.* [47] screened, via agar diffusion disk assay, dichloromethane, methanol and water extracts of cultivated seaweed for their activities against *Aeromonas salmonicida* ssp*. salmonicida*, *A. hydrophila* ssp. *hydrophila*, *Yersinia ruckeri*, *Pseudomonas anguilliseptica* and *Vibrio anguillarum*. These latter two were the more susceptible strains to dichloromethane extracts of *Asparagopsis armata*, *Ceramium rubrum*, *Drachiella minuta*, *Falkenbergia rufolanosa*, *Gracilaria cornea* and *Halopitys incurvus*. Crude extract of *Asparagopsis* sp. also showed *in vivo* antibacterial activity against shrimp *Vibrio* pathogens [62].

Dubber and Harder [55] investigated antibacterial effects of hexane and methanol extracts of *Mastocarpus stellatus*, *Laminaria digitata* and *Ceramium rubrum* on fish pathogenic bacteria. Gram-positive *Bacillaceae* were generally more susceptible than Gram-negative *Vibrionaceae*, *Listonella anguillarum*, *Pseudomonas anguilliseptica* and *Aeromonas salmonicida*.

Cavallo *et al.* [45] confirmed the activity of chloroform/methanol extracts against some pathogenic *Vibrio* species using the disk diffusion method. The best candidates were *G. longissima* and *C. linum*, active against *V. alginolyticus*, *V. vulnificus* and *V. ordalii*.

Cortés *et al.* [130] evaluated the antimicrobial activity of *Ceramium rubrum* from Chile on the bacteria *Yersinia ruckeri* and the oomycete *Saprolegnia parasitica*, causing enteric red mouth disease and saprolegniasis, respectively. The ethanol and dichloromethane extracts were effective against *S. parasitica* using the agar dilution method, and MIC values were determined by the broth dilution method. The whole extract was more active than the individual components, suggesting a synergistic effect among the components.

García-Bueno *et al.* [53] observed by the microplate method that water extracts of *Grateloupia turuturu*, from the French Atlantic coast, were active against the abalone pathogen *V. harveyi* strain ORM4. However, extracts from *Palmaria palmata* collected in the same coast were inactive.

Krish and Das [68] reported that the crude methanol, ethanol and ethyl acetate extracts of *Cladophora rupestris* collected in a Mediterranean area, showed good antimicrobial activity against *V. harveyii*, *V. parahaemolytical* and *V. alginolyticus*, which were measured with the agar diffusion method and the zone of inhibition. The fatty acid profile showed palmitic, myristic, oleic, alpha linolenic, palmitoleic and linoleic acids. Although scarcer, studies with other algal components have been reported. Tanniou *et al.* [70] tested the antibacterial activity of phenolic products from *Sargasum* against *Vibrio aestuarianus*, *V. anguillarum* and *V. parahaemolyticus*.

In addition to *in vitro* screenings, studies have considered the antimicrobial effects incorporating the seaweed extracts either in the feeds or added into the water in which the fish and shrimp were reared. It has been suggested that the direct addition of antimicrobial seaweed substances into the water culture led to interferences between pathogens, rather than their growth. In their recent review, Vatsos and Rebours [14] emphasized on the need of pharmacodynamics and pharmacokinetics studies and also on the need of studies confirming their applicability on a commercial scale.

Thanigaivel *et al.* [50] confirmed that the ethanol extracts of *Chaetomorpha antennina in vitro* by the agar well diffusion method and *in vivo* on shrimps infected with *Vibrio parahaemolyticus.* The ethanol extract of *C. antennina* was very effective in controlling *V. parahaemolyticus*, which is resistant to ampicillin and sensitive to erythromycin. Thanigaivel *et al.* [9,50,51,131] confirmed *in vitro* the antibacterial activity of aqueous and ethanol extracts of *Padina gymnospora*, *Gracilaria folifera*, *Sargassum cinereum* and *S. longifolium* via the agar well diffusion method and *in vivo* in fingerlings of *Oreochromis mossambicus* (tilapia) infected with *Pseudomonas aeruginosa* and detection by PCR.

Oliveira *et al.* [132] performed a 40-day experiment consisting of the inclusion of *Ascophyllum nodosum* meals at a dose of 20 g/kg on the diet of *Nile tilapia* inoculated with *Aeromonas hydrophila*. The width was greater for the treatment with the algal meal, but there was no influence on the performance parameters of the fingerlings. The occurrence of lesions in animals inoculated with *A. hydrophila* and fed with the alga was lower and declined in a shorter period of time than in the control group; prevention of hepatopancreatic congestion in infected animals was also observed.

### 5.3. Human Health

#### 5.3.1. Antibacterial and Antifungical

The potential of seaweed as a source of compounds active against pathogenic microorganisms has been confirmed in different studies. Padmakumar and Ayyakkannu [69] screened 80 species against bacterial and fungal pathogens. Of the algae, 70% exhibited antibacterial activity but only 27.5% showed antifungal activity. Among the species tested, *S. aureus*, *Vibrio spp* and *Trichophyton mentagrophytes* were the most susceptible, whereas *P. aeruginosa* and *Aspergillus flavus* were the most resistant.

Freile-Peligrin and Morales [63] evaluated the activity against Gram-positive, Gram-negative and fungus of the ethanolic and lipid-soluble extracts from seaweed species from the coast of Yucatan, Mexico, and found that more than 80% species were active against the bacteria Gram-positive tested. Ely *et al.* [42] tested methanolic extracts of Indian seaweed against clinical bacteria and fungi, using paper disk assays. *Stoechospermum marginatum* extract was effective only against *Vibrio cholerae* and *Cladophora prolifera* against *V. cholerae*, *Klebsiella* sp., *S. aureus* and *Aspergillus niger*.

Kim *et al.* [123] reported antimicrobial activitiy of crude extracts and solvent fractions from *Sargassum muticum*, especially the dichloromethane fraction against *B. subtilis*, *Listeria monocytogenes*, *S. aureus*, *E. coli*, *Salmonella enteritidis* and *P. aeruginosa.*

Dhanya et al. [133] reported the antimicrobial activity of extracts from *Ulva reticulata* against *Staphylococcus aureus*, *Escherichia coli*, *Pseudomonas aeruginosa*, *Salmonella typhi*, and *Bacillus subtilis*. 

Kim *et al.* [134] reported the *in vitro* broad-spectrum antimicrobial activity of ethyl-ether extracts of *U. lactuca* against methicillin-resistant *S. aureus* (MRSA), but not antifungal activity against *C. albicans*. Muñoz-Ochoa *et al.* [86] screened sixty ethanol extracts of marine flora of Baja California Sur (Mexico) to evaluate the reversing effect of the bacterial resistance to antibiotics in combination with a sublethal concentration of ampicillin or erythromycin. Thirthy five of the assayed extracts showed inhibitory activity against *S. aureus*, 48 were active against *Streptococcus pyogenes*, but none were active against *E. coli*. The most active extracts were from *Laurencia* spp., *Gelidium robustum*, *Chnoospora implexa*, *Padina mexicana*, *Gracilaria subsecundata* and *Dictyopteris undulata.*

Oh *et al.* [135] tested for antimicrobial activity against bacteria and fungi human pathogens the bromophenols isolated from the red alga *Odonthalia corymbifera*, and found that natural products were more active against fungi and synthetic bromophenols against bacteria. Shanmughapriya *et al.* [52] tested 40 different seaweeds; of these, seven species showed high antimicrobial activity against multirresistant pathogens.

Osman *et al.* [82] observed the antimicrobial activities of crude extracts from the species of Rhodophyta, Chlorophyta and Phaeophyta against *B. subtilis*, *S. aureus*, *E. coli*, *Klebsiella pneumoniae* and *Candida albicans*. Taskin *et al.* [92] reported inhibition of *C. albicans* growth by a methanolic extract from *Cystoseira mediterranea*.

Manilal *et al.* [88] tested the crude methanolic extracts of marine organisms against multiresistant human pathogens. They found that the Falkenbergia-phase of *Asparagopsis taxiformis* was highly active, and the most abundant metabolites were oleic acid and *n*-hexadecanoic acid.

Pierre *et al.* [98] reported *Chaetomorpha aerea* extracts in methanol and in water exhibited *in vitro* antimicrobial activity against three Gram-positive bacteria. Salem *et al.* [118] screened methanolic and ethyl acetate extracts from eight different seaweeds collected from Hurghada, a city on the Red Sea coast in Egypt, for their antibacterial activities, evaluating the zone of inhibition and MIC values. Higher susceptibility of Gram-positive bacteria to the algal extracts was observed, and ethyl acetate extract of *C. racemosa* was the most inhibitory extract.

Adaikalaraj *et al.* [113] evaluated the antibacterial activity of methanol and aqueous extracts from *Gracilaria verrucosa*, *G. ferugosoni*, *Hypnea musciformis*, *Enatiocladia prolifera* and *Gelidium* species against selected bacterial pathogens and one yeast by using the disc diffusion method. In most of the seaweeds, methanol extract was found to be more effective. *Salmonella typhi*, *S. aureus*, *B. subtilis* and *C. albicans* were resistant to all aqueous extracts. Águila-Ramírez *et al.* [3] confirmed that ether extracts of *L. johnstonii*, *D. flabellata* and *U. lactuca* from the Gulf of California showed activity against *Staphylococcus aureus*, but only the buthanol fraction from *D. flabellata* was active. No activity was observed against *E. coli*, *Bacillus cereus*, *B. subtilis* and *Staphylococcus epidimidis*. Amorim *et al.* [90] determined the effect on bacterial growth of a crude sulfated polysaccharide from *G. ornate* and fractions extracted at different temperatures, showing low contents of sulfate (5.88%–10.3%) and proteins (0.1%–3.7%). Go-3 was tested on the growth of bacteria (*B.subtilis*, *S.aureus*, *Enterobacter aerogens*, *E. coli*, *P. aeruginosa*, *Salmonela choleraesuis* and *S. typhi*), but only *E. coli* was inhibited. Rosaline *et al.* [78] screened the antibacterial efficacy of extracts in hexane, ethyl acetate, acetone and methanol of *Sargassum wightii*, *Chaetomorpha linum* and *Padina gymnospora* against selected Gram-positive and Gram-negative human pathogenic bacteria. The acetone extracts of the three algae showed higher inhibitory activity for the selected bacterial species than other solvent extracts. *P. gymnospora* and *S. wightii* were more active than *C. linum* against human pathogenic bacteria using the disc diffusion method. Águila-Ramírez *et al.* [3] reported antibiosis of the extracts from *L. johnstonii*, *U. lactuca* and *D. flabellata*. The ether extracts were more active in comparison with butanol extracts against *S. aureus*. *L. johnstonii* extracts showed good activity against *E. coli*. Mhadhebi *et al.* [46] screened 24 organic fractions of 6 seaweeds from the Tunisian Mediterranean coast against 8 human pathogenic bacteria and 5 *Candida* pathogenic strains. All the extracts exhibited moderate to weak activity against *S. aureus; S. epidermis*, *E. coli* and *Micrococcus luteus*. The chloroform and the ethyl acetate extracts from *Cystoseira crinita* and *C. sedoides* showed a higher antifungal activity against four *Candida* strains.

Bianco *et al.* [94] evaluated the *in vitro* anti-infective potential of organic extracts from different marine organisms, including seaweed collected along the Brazilian coast, and found that *Osmundaria obtusiloba* showed moderate activity against *P. aeruginosa.* Genovese *et al.* [136] confirmed the antifungal activity of *Asparagopsis taxiformis*, from the Straits of Messina (Italy), against *Aspergillus fumigatus*, *A. terreus* and *A. flavus*. The lowest MIC observed were <0.15 mg/mL, and the highest were >5 mg/mL. Jassbi *et al.* [114] reported antioxidant and antifungal activity in the water extracts of a red algae, *Hypnea flagelliformis*, and two brown algae, *Cystoseira myrica* and *Sargassum boveanum*, from the Persian Gulf. The active substances against *S. aureus* and *B. subtilis* using the agar disc diffusion and nutrient broth micro-dilution bioassays were identified as free fatty acids, fucosterol, cholesterol and 22-dehydroxychlosterol. Mathan *et al.* [129] assayed acetone/methanol extracts further fractionated with ethyl ether and with butanol, in an agar diffusion test against human pathogenic bacteria: *E. coli*, *S. aureus*, *B. cereus*, *B. subtilis* and *S. epidermidis*. Mohandass *et al.* [137] proposed the use of *Sargassum cinereum* extracts as a reducing agent in the extracellular synthesis of silver nanoparticles. The MIC against *Staphylococcus aureus* was 2.5 μL (25 μg/disc), and against *Enterobacter aerogenes*, *S. typhi* and *Proteus vulgaris* were 100 μg/disc. Osman *et al.* [138] tested macroalgae collected from Egypt against pathogenic Gram-positive and Gram-negative bacteria, and one clinical yeast strain, *Candida albicans*. The tested species of Chlorophyta were more potent inhibitors than those from Rhodophyta and Phaeophyta. The extract of *U. fasciata* was the most active (phthalate esters derivatives being the active components), followed by *E. compressa*, *U. lactuca* and *E. linza*; *K. pneumoniae* was the most sensitive microorganism. Saritha *et al.* [79] reported antibacterial activity of *U. lactuca* extracts against *Shigella sonnei*, *B. subtilis*, *E. feacalis*, *S. typhimurium*, *E. coli*, S*. aureus*, *S. pyogenes* and *Staphylococcus epidermis.*

Al Hazzani *et al.* [84] reported higher *in vitro* antimicrobial activity of methanol and acetone extracts from the brown algae (*L. japonica*, *U. pinnatifida*, *E. bicyclis*) than from the red alga *P. tenera* against Gram-positive and Gram-negative bacteria, some were antibiotic-resistant such as methicillin-resistant *S. aureus* and *P. aeruginosa*, and, resistant against yeast, *C. albicans*. Horincar *et al.* [85] studied macroalgae from the Romanian Black Sea coast for their volatile compounds content. The major components in the extract of *Ceramium virgatum* were 3-hexen-2-one, acetone, hexanal and *o*-cymene, in that of *Cladophora vagabunda* were hexanal, octane, nonanal, octanal, 2,5,5-trimethyl-2-hexene, 3-hexen-2-one, and *o*-cymene and in that of *U. intestinalis* were hexanal, trichloromethane, nonanal, 3-hexen-2-one, and octanal, and had a greater content of mono- and polyunsaturated fatty acids, palmitic acid, arachidonic acid, and oleic acid. The lipid fatty acids affected the biological activity against *S. enteritidis*, *E. coli*, *Listeria monocytogenes*, *B. cereus*, and differences among algal families were observed, *i.e.*, the total lipids from Laminariaceae were more active antimicrobial than those from Alariaceae [139]. Ismail *et al.* [125] reported the antibacterial and antifungal activities of aqueous extracts and fractions of *Zonaria tournefortii* against *C. albicans*, *Cryptococcus neoformans*, and several multi-resistant *S. aureus*. Jaswir *et al.* [81] produced crude extracts from four species of brown seaweed (*Sargassum plagyophillum*, *S. flavellum*, *S. binderi* and *Padina australis*) from Malaysia. Crude extracts showed no antimicrobial activity against the Gram-negative bacteria (*P. aeruginosa* and *E. coli*), whereas the Gram-positive bacteria (*B. subtilis* and *S. aureus*) were inhibited. Kavita *et al.* [83] screened the methanol extract of 38 seaweed samples against human pathogenic bacteria, and Gram-positive (*S. aureus* and *B. subtilis*) and Gram-negative (*E. coli* and *P. aeruginosa*) bacteria. *Laurencia papillosa* showed maximum antimicrobial activity, and the active fraction was identified as a cholesterol derivative, 24-propylidene cholest-5-en-3 beta-ol. The MIC values against clinical isolates ranged from 1.2 to 1.7 µg/mL. Krish and Das [68] reported that the crude methanol, ethanol and ethyl acetate extract of *Cladophora rupestris* at 250–1000 µg/mL showed concentration-dependent antimicrobial activity against several human pathogenic bacteria, including *E. coli*, *P. aeruginosa* and *S. aureus*. The activity was evaluated with the zone of inhibition, which was compared to that of amphicilin. Whereas the methanol extract was more active against *E*. coli, the ethyl acetate was a greater inhibitor of *S. aureus* and the ethanol extract against *S. aureus* and *P. aeruginosa.* Nogueira *et al.* [110] reported modulation of antibiotic activity between the *Padina sanctae-crucis* ethanolic and methanolic extracts and *E. coli* and *S. aureus*, and a moderate modulatory effect against these microorganisms and *P. aeruginosa*, *Candida tropicalis* and *C. kruseim.* Stabili *et al.* [65] confirmed the activity against human pathogenic bacteria and yeasts of lipid extracts of one Mediterranean alga with the difussion disk method. Tanniou *et al.* [70] observed the antibacterial activity of phenolics from *Sargasum* collected in different countries against *E. coli*, *S. aureus* and *P. aeruginosa*.

El Wahidi *et al.* [140] reported the antimicrobial activity of dichloromethane and ethanol extracts from seaweeds collected from the Moroccan’s Atlantic coast against 2 Gram-positive (*B. subtilis and S. aureus*) and 2 Gram-negative (*E. coli* and *P. aeruginosa*) bacteria, and against two pathogenic yeasts (*Candida albicans* and *Cryptococcus neoformans*) using the agar disk-diffusion method. *Cystoseira brachycarpa*, *C. compressa*, *Fucus vesiculosus*, and *Gelidium sesquipedale* showed better antimicrobial activity with a broad spectrum.

Karthikeyan *et al.* [80] tested the antibacterial activity for organic solvent extracts from seaweeds from Kodinar coast in India: *Enteromorpha* sp., *Cystoseria indica*, *Sargassum swartzii*, *Gracilaria corticata*, *Caulerpa taxifolia* and *C. racemosa* against *E. coli*, *Proteus* sp., *P. aeruginosa*, *Klebsiella pneumoniae* and *S. aureus*. The maximum antibacterial activity was observed in the ethanol extracts of all the seaweeds except *C. racemosa*. Kolsi *et al.* [44] evaluated the antibacterial and antifungical activities of organic extracts (hexane, ethylacetate and methanol) of 13 seaweed species from the Tunisian coastline against 8 Gram-negative and Gram-positive human pathogens, yeast and a fungi in an agar disk diffusion test. They found that brown algae showed higher inhibitory activity on bacteria than on fungi. Kosanic *et al.* [101] reported that the acetone extracts of *U. lactuca* and *E. intestinalis* showed antimicrobial and cytotoxic activity on four human cancer cell lines. The extracts of *U. lactuca* showed MIC values ranging from 0.156 to 5 mg/mL, but it was relatively weak in comparison to standard antibiotics. *Bacillus mycoides* and *B. subtilis* were the most susceptible to the tested extracts. Lopes *et al.* [141] proposed phlorotannin extracts due to their antifungal activity against several yeast and dermatophyte strains, using a micromethod for the evaluation of the MIC and the MLC. Pinteus *et al.* [43] evaluated the methanol, *n*-hexane and dichloromethane extracts of macroalgae (Rhodophyta, Chlorophyta and Heterokontophyta divisions) from Peniche coast (Portugal) for antibacterial activity, evaluated by the disc diffusion method against *B. subtilis* and *E. coli*. The antifungal potency of the *Sphaerococcus coronopifolius* extracts, evaluated against *Saccharomyces cerevisiae* as a model, was similar to the standard amphotericin B. *Asparagopsis armata* and *Sphaerococcus coronopifolius* revealed the most potent antimicrobials. Wei *et al.* [142] found that an anthraquinone dihydroxy derivative, 1,8-dihydroxy-anthraquinone, first isolated from *Porphyra haitanensis*, showed antibacterial activity against *S. aureus*, inhibiting cell growth at logarithmic phase due to its interaction with the cell wall and cell membrane, leading to increased permeability, leakage of cytoplasm and the deconstruction of cell.

Patra *et al.* [103] reported that *Enteromorpha linza* L. essential oil, containing high amounts of acids (54.6%) and alkenes (21.1%), was effective against both *E. coli* and *S. typhimurium*. MIC and MBC values for both pathogens were 12.5 mg/mL and 25.0 mg/mL, respectively. Essential oil induced a bactericidal effect via structural membrane damage caused by its deposition in the cytosol or through enzymatic degradation of bacterial intracellular enzymes that resulted in cellular lysis.

Spavieri *et al.* [59] tested the antimycobacterial activity of crude extracts of 21 species of brown algae from British and Irish waters but only the *Bifurcaria bifurcata* extract was weakly active against *Mycobacterium tuberculosis*.

Marine algae could be a source of therapeutic agents for chronic gastritis and peptic ulceration. Lee *et al.* [143] screened 27 Korean species of seaweed for potential anti-*Helicobacter pylori* activity, and seven showed strong inhibitory activity based on the agar diffusion method. The strongest activity was observed for ethanol extracts from *Ishige okamurae*. The inhibition zone of this extract was 9.0 mm at 1 mg/disk, the MIC was 12 µg/mL based on the broth microdilution assay, and the free urease assay confirmed that the 80% methanol extracts had 75.4% inhibition at 0.1 mg/mL. Both the *I. okamurae* phenolic compounds and the nitrogen compounds of the extract significantly inhibited *H. pylori*. No toxicity was observed in a study with BALB/c mice at a dose of 5 g/kg body weight.

Ha *et al.* [100] screened 44 seaweed species for anti-*Gardnerella vaginalis* activity and 27 of them showed antimicrobial activity by the agar disk-diffusion method. *Ulva pertusa* exhibited the highest activity against the strains of *G. vaginalis* causing vaginosis (MIC = 312 µg/mL), and against *Candida albicans*, the main cause of candidiasis (MIC = 2.5 mg/mL). Nitrogenous compounds were the main active agents against *G. vaginalis*. No inhibitory effect against *Lactobacillus gasseri* and *Lactobacillus jensenii*, present in the normal vaginal microflora, were observed at 10 mg/mL.

Vedhagiri *et al.* [144] evaluated compounds isolated from *Asparagopsis taxiformis* for their inhibitory action against *Leptospira javanica* isolated from rodent carriers and found MICs and MBC in the range of 100–1600 μg/mL, whereas those for penicillin and doxycyline were in the range of 25–200 μg/mL. The GC-MS analysis revealed the presence of 4,5-dimethyl-1*H*-pyrrole-2-carboxylic acid ethyl ester, fatty acids, 14-methyl-pentadecanoic acid methyl ester, octadecanoic acid methyl ester, octadec-9-enoic acid 2,3-dihydroxy-propyl ester, 9-octadecanoic acid, methyl ester and trace amount of chlorobenzene.

##### Acne

Effective and safe acne vulgaris therapies could be derived from algal extracts. Amiguet *et al.* [105] observed that the crude ethyl acetate extracts from *Fucus evanescens* showed strong antibacterial activity against *Propionibacterium acnes* (culture collection and clinical isolate), and also against *Hemophilus influenzae*, *Legionella pneumophila*, and *Streptococcus pyogenes*, *Clostridium difficile* and methicillin-resistant *S. aureus*, whereas *B. cereus*, *E. coli*, *K. pneumoniae*, and *P. aeruginosa* were not significantly affected. The main active compound was identified as a β-d-galactosyl *O*-linked glycolipid.

Lee *et al.* [104] confirmed the efficacy of a crude methanolic extract from *Eisenia bicyclis*. The ethyl acetate fraction showed the strongest antibacterial activity, the most active compound among the six isolated from this fraction was fucofuroeckol-A, with a MIC value ranging from 32 to 128 µg/mL. This compound also reversed the erythromycin and lincomycin resistance of *P. acnes*. A weak synergistic effect of fucofuroeckol with erythromycin and with lincomycin was observed.

##### Oral Microbes

Periodontitis is a chronic inflammatory disease initiated by Gram-negative pathogens, such as *Prevotella intermedia* and *Porphyromonas gingivalis*. Traditional antibiotic therapies for periodontitis target the bacterial infection, but identification of new therapeutic agents with few or no side effects and potent antimicrobial activity is desirable.

*Enteromorpha linza* extracts displayed antimicrobial activity against *P. intermedia* and *P. gingivalis* without side effects at a moderate dose [145] and in a mouth rinse has shown effects similar to those of Listerine^®^ against gingivitis. Park *et al.* [102] reported that the main active compounds, isolated by Sephadex LH-20 gel and reverse-phase HPLC, were unsaturated fatty acids, stearidonic acid (SA) and gamma-linolenic acid (GLA). Their MIC values were 39.06 µg/mL against *P. intermedia* and 9.76 µg/mL against *P. gingivalis*, respectively. The MIC values of pure compounds against *Candida albicans* were 312.50 μg SA/mL, 78.12 μg GLA/mL, 312.50 μg triclosan/mL and 4.88 μg chlorhexidine/mL, and against *Streptococcus mutans* were 1250 SA μg/mL, 625 μg GLA/mL and 9.76 μg chlorhexidine/mL [145].

Kim *et al.* [146] confirmed the antimicrobial activity of ethanol extracts of *L. japonica* against oral microbials. The MICs of ethanol extracts were 250 and 62.5 µg/mL against *Actinomyces naeslundii* and *Actinomyces odontolyticus*, respectively, and 250 and 62.5 µg/mL for *Fusobacterium nucleatum* and *Porphyromonas gingivalis*, respectively. The MBCs of *A. naeslundii* and *A. odontolyticus* were 500 and 250 µg/mL, respectively. A dose dependent effect and a change in the cell surface texture of *Streptococcus mutans*, *A. odontolyticus* and *P. gingivalis* was observed*.*

The topical application of products derived from *n*-3 fatty acids can protect against inflammation-induced tissue and bone loss associated with periodontitis in experimental models [147]. The safety of stearidonic acid was confirmed in rat and in human studies [102]. Lee *et al.* [148] reported, via the broth dilution method and checkerboard and time-kill assay, that fucoidan was efficient against oral bacteria, either alone or with antibiotics MIC/MBC values for the fucoidan against oral bacteria ranging between 0.125–0.50/0.25–1.00 mg/mL, for ampicillin 0.125–64/0.5–64 μg/mL and for gentamicin 2–256/4–512 μg/mL, respectively. The MIC and MBC values were reduced to one sixteenth and the rate of killing was increased when fucoidan was combined with antibiotics.

#### 5.3.2. Antiprotozoals

De Felício *et al.* [124] reported that some fractions from *Bostrychia tenella* were more active than gentian violet and amphotericin B as antiprotozoal agents against *Trypanosoma cruzi* and *Leishmania amazonensis.* Among the volatile compounds identified were fatty acids, low molecular mass hydrocarbons, esters and steroids and also uncommon substances, such as neophytadiene. Crude extracts of 21 brown algae were screened by Spavieri *et al.* [59] against *Trypanosoma cruzi* and *T. brucei rhodesiensi* and *Leishmania donovani*. All algal extracts showed significant activity against *T. brucei rhodesiensi* and *L. donovani*, and two Sargassaceae strains were the most potent. Bianco *et al.* [94] reported that the organic extracts from *Anadyomene saldanhae*, *Caulerpa cupressoides*, *Canistrocarpus cervicornis*, *Dictyota* sp., *Ochtodes secundiramea*, and *Padina* sp. collected along the Brazilian coast showed promising antiprotozoal results against *Leishmania braziliensis*, and only *Dictyota* sp. was effective against *T.cruzi.*

#### 5.3.3. Antivirals

Yasuhara-Bell and Lu [11] reviewed the antiviral properties of marine organisms, including those from algae, active against a wide range of viruses, including herpes viruses (HSV-1, HSV-2, HCMV), togaviruses (Sindbis virus, Semliki Forest virus), paramyxoviruses (RSV), rhabdoviruses (VSV), and both human and simian immune deficiency viruses (HIV and SIV). Extracts and different compounds from algae might be promising for future antiviral design. Extracts of *Gigartina atropurpurea*, *Plocamium cartilagineum*, *Splachnidium rugosum*, and *Undaria pinnatifida* inhibited *Herpes simplex* virus types 1 (HSV-1) and 2 (HSV-2) [149]. Bouhlal *et al.* [60] screened fifty-five aqueous, methanolic, chloroform-methanolic and dichloromethanolic extracts of Rhodophyta from the coast of Morocco for inhibitory compounds against HSV-1 by the cell viability method. The aqueous extracts of 10 seaweeds were capable of inhibiting the replication of HSV-1 *in* *vitro* at an EC_50_ ranging from <2.5 to 75.9 µg/mL, without any cytotoxic effect on the Vero cells in the range of the concentrations assayed.

Different chemical structures were responsible for this activity. The glycolipid sulfoquinovosyldiacylglycerol from *Osmundaria obtusiloba* collected from Southeastern Brazilian coast demonstrated antiviral activity against HSV-1 and HSV-2, with EC_50_ values of 42 μg/mL and and 12 μg/mL, respectively [10,150]. Koishi *et al.* [151] reported that extracts from *Caulerpa racemosa*, *Canistrocarpus cervicornis*, *Padina gymnospora* and *Palisada perforate* attenuated dengue virus infection probably by acting at an early stage of the cycle of infection, like binding or internalization.

The diterpenes 8,10,18-trihydroxy-2,6-dolabelladiene and (6*R*)-6-hydroxydichotoma-4,14-diene-1,17-dial, isolated from *Dictyota pfaffii* and *D. menstrualis*, respectively, inhibited HSV-1 infection in Vero cells [152]. Mendes *et al.* [153] reported that the extract of the marine algae *Stypopodium zonale*, two meroditerpenoids (atomaric acid and epitaondiol) derived from it, and a methyl ester derivative of vatomaric acid exerted antiviral activity against human metapneumovirus replication with a selectivity index of 20.78, >56.81, 49.26, and 12.82, respectively.

Lectins, polysaccharides, and tannins with anti-HIV-1 activity have been isolated from marine macroalgae [154]. The antiviral activities of sulfated polysaccharides from seaweed, their structural chemistry and their potential for therapeutic application, have been reviewed [29,61,115,155]. Marine polysaccharides can inhibit the replication of viruses through interfering with a few steps in the virus’ life cycle or improving the host immune responses to accelerate the process of viral clearance. Naesens *et al.* [156] reported that a 26 kDa xylogalactofucan from *Sphacelaria indica*, containing (1→3)-linked l-fucopyranosyl and d-galactopyranosyl residues and rich in sulfated polysaccharides, showed strong activity against the human herpevirus-6 (HHV-6). Bandyopadhyay *et al.* [157] reported that the 21 kDa alginate from *S. indica* (41% guluronic and 59% mannuronic acid) displayed IC_50_ values of 0.6–10 μg/mL against HSV-1, and attributed the inhibition to direct interaction of polysaccharides with viral particles. Fucoidan from *Cladosiphon okamuranus* was stronger than ribavirin against Newcastle disease virus in the Vero cell line at the early stages of infection, by inhibition of viral penetration [158]. Polysaccharides from Rhodophytas had major antiviral activity, whereas those from Phaeophytas showed major antitumoral and other activities diversity.

Carragenans of the red algae were active against both enveloped and non-enveloped viruses. The specific antiviral effect was related to the molecular weight and degree of sulfation, and different viruses depended on the type of carrageenan, viral serotype and the host cell. The number of necrotic lesions on Xanthinc tobacco leaves was lessened by concurrent inoculation with tobacco mosaic virus and kappa/beta-carrageenan from *Tichocarpus crinitus*, compared to virus-inoculated leaves [159]. Carrageenans from *Meristiella gelidium* were effective inhibitors of dengue virus DENV-2 if compared to *G. griffithsiae* samples and reference polysaccharides [160].

Jiang *et al.* [89] reported the *in vitro* antioxidant, antimicrobial and antischistosomal activities of the pethroleum ether fraction of a *Grateloupia livida* ethanolic extract. This fraction showed antimicrobial activity against the human pathogenic trematode *Schistosoma japonicum* adult worm. Neither cellular cytotoxicity at concentrations up to 100 μg/mL nor acute oral toxicity in mice at doses up to 2000 mg/kg was observed. Most of the 25 components identified have known antioxidant and antimicrobial activities.

### 5.4. Food

Microbial food spoilage is responsible for important economic losses due to the ubiquitous presence of microorganisms and their impact on human health. Food products also undergo autoxidation, leading to the development of rancid flavors and odors, and a reduction in the sensory attributes, nutritional quality and food safety. Replacement of synthetic additives and exploitation of new natural antioxidant and antibacterial substances with a possible role as nutraceutical agents is encouraged. Multiple functionality seaweed extracts could be incorporated into foods as natural preservatives to enhance the food quality, safety and stability [33,54,161].

Different studies confirmed the antimicrobial action of seaweed against food spoilage microorganisms. Phlorotannins are potent antioxidants and showed a strong bactericidal effect against food-borne bacteria [15]. The highest antimicrobial action is not necesarily coincident with the highest antioxidant action. Devi *et al.* [162] evaluated ten edible seaweeds from India for antioxidant and antimicrobial activity against food-borne pathogens. *Gelidella acerosa* and *Haligra* sp have high phenolic content and exhibited antibacterial activity against *S. aureus*, but *G. acerosa* has the highest antioxidant activity. Krish and Das [68] reported that the antioxidant and antimicrobial activity of *Cladophora rupestris* solvent extracts and *Scytosiphon lomentaria* methanol extract inhibited the Gram-negative bacterium *S.* *typhimurium* [92].

Cox *et al.* [6] tested *Laminaria digitata*, *L. saccharina*, *Himanthalia elongata*, *Palmaria palmata*, *C. crispus* and *Enteromorpha spirulina* from the Irish coast using a microtiter method against food spoilage and food pathogenic bacteria: *L. monocytogenes*, *S. abony*, *E. faecalis* and *P. aeruginosa*. All methanolic extracts inhibited the bacterial growth except *C. crispus* extracts. Brown algal extracts showed more antibacterial activity than red and green extracts, and *H. elongata* was the most efficient. Cox *et al.* [54] found that the hydrophilic extract from *H. elongata* provided up to 100% inhibition of *S. abony* and L. monocytogenes. Concentration-dependent antibacterial activity was observed in the range of 0.125–8 mg/mL, but no inhibition was detected under 1 mg/mL. The extracts showed bactericidal effect in carbohydrate model food systems and bacteriostatic effect in protein model food systems (at 1%–10%). Sodium benzoate exhibited greater inhibition at 2–0.5 mg/mL and showed bacteriostatic effect in carbohydrate medium, whereas the extract had a bactericidal effect.

Gupta *et al.* [117] reported that extracts from *H. elongata* at 6% inhibited the growth of food spoilage microorganisms (*P. aeruginosa* and *E. faecalis*) and food pathogens (*L. monocytogenes* and *S. abony*). Lower concentrations of extract prolonged the lag phase and reduced both the exponential growth rate and final population densities of the culture.

Beaulieu *et al.* [163] measured the antibacterial activity of a protein extract from *Saccharina longicruris* and from the <10 kDa and >10 kDa fractions via liquid growth inhibition assays using an optimized critical microdilution method against *E. coli*, *Micrococcus luteus*, *Brochotrix thermosphacta*, *E. faecalis*, *Mycobacterium smegmatis*, *S. aureus*, *Streptococcus*, *P. aeruginosa*, *Vibrio splendidus*, and *V. gigantis*. No activity was detected when each peptide was tested separately at 2.50 mg/mL, but significant activity was observed when mixed all together at this total peptide concentration.

Boisvert *et al.* [56] tested *S. longicruris*, *Ascophyllum nodosum* and *U. lactuca* from the St. Lawrence Estuary against *E. coli*, *M. luteus* and *B. thermosphacta*, determining the MIC values using a broth microdilution assay. *U. lactuca* extracts obtained with pressurized ethanol showed the highest antibacterial activity against the strains tested, and was therefore more effective towards Gram-negative bacteria, whereas *A. nodosum* was more efficient towards Gram-positive strains. The higher metal ions content in *U. lactuca* extracts (Al, Fe, Co, Ni, Cu, Zn, Cd, Hg, Pb) than in the other extracts could be determinant for the activity.

Moronery *et al.* [164] added a spray-dried *Laminaria digitata* extract (9.3% laminarin, 7.8% fucoidan) to minced pork patties at levels of 0.01%–0.5% (*w*/*w*). After storage in a modified atmosphere for 14 days at 4 °C, at 0.5% the extract exerted the greatest lipid pro-oxidant activity in fresh patties, whereas the lipid oxidation was decreased in cooked patties, but the microbiological status, pH, and water-holding capacity were not influenced.

Shiahaan *et al.* [165] prepared *S. japonica* as a powder (500–900 µm) for the physical adsorption and further release of allyl isothiocyanate, an inhibitor of food-borne bacteria. When the alga was deoiled, higher adsorption of allyl isothiocyanate was observed in comparison to raw alga. No loss of activity was detected against *E. coli*, *S. typhimurium* or *B. cereus*, and only a nominal activity against *S. aureus* was observed.

### 5.5. Other Uses

Other novel or less common applications have also been proposed based on the antimicrobial properties of seaweed extracts and compounds. Macroalgae contain compounds which could be suited for controlling specific plant pathogens. Jiménez *et al.* [166] studied *in vitro* and *in vivo* aqueous and ethanolic extracts obtained from nine Chilean marine macro-algae collected at different seasons for properties that reduce the growth of plant pathogens or decrease the injury severity of plant foliar tissues following pathogen infection. Manilal *et al.* [109] prepared methanol extracts from *Lobophora variegata* of the Kollam coast and confirmed that the *in vitro* antimicrobial activity of algal extract was investigated in Petri dishes. The extracts showed higher toxicity against bacterial pathogens than against mosquito pupae, nematodes and plant seeds.

Hierholtzer *et al.* [167] studied the effect of phloroglucinol and phenolic compounds extracted from *Laminaria digitata* on mixed microbial anaerobic organisms used for anaerobic digestion of algae. The bactericidal activity of phlorotannins is a function of their level of polymerization. Phlorotannins induce significant extra- and intra-cellular effects, with the disruption of cell membranes observed with most microorganisms.

Marine macroalgae polysaccharides could be prebiotic ingredients for both human and animal health applications. Prebiotics are non-digestible compounds that stimulate the growth and/or activity of beneficial gut microbiota which, in turn, confer health benefits on the host. Feeding with algal polysaccharides can modify the fecal and colonic population of *Lactobacillus* spp., *Bifidobacterium* and *Enterobacterium* [168].

Tan *et al.* [95] developed an antimicrobial wound dressing, consisting in a hydrogel prepared with polyvinyl alcohol, polyvinylpyrrolidone and 1% seaweed extract. It was effective against nine clinically-relevant wound pathogens as the commonly used silver-based dressing, *Acticoat*. The dressing, evaluated using agar diffusion tests, gel leaching and gel transfer assays, proved effective up to 97 h, without leaving a residue following five subsequent transfers. Antimicrobial activity was stable for up to 6 months of storage at 4 °C, but activity was slightly reduced after 15 weeks.

**Figure 3:**
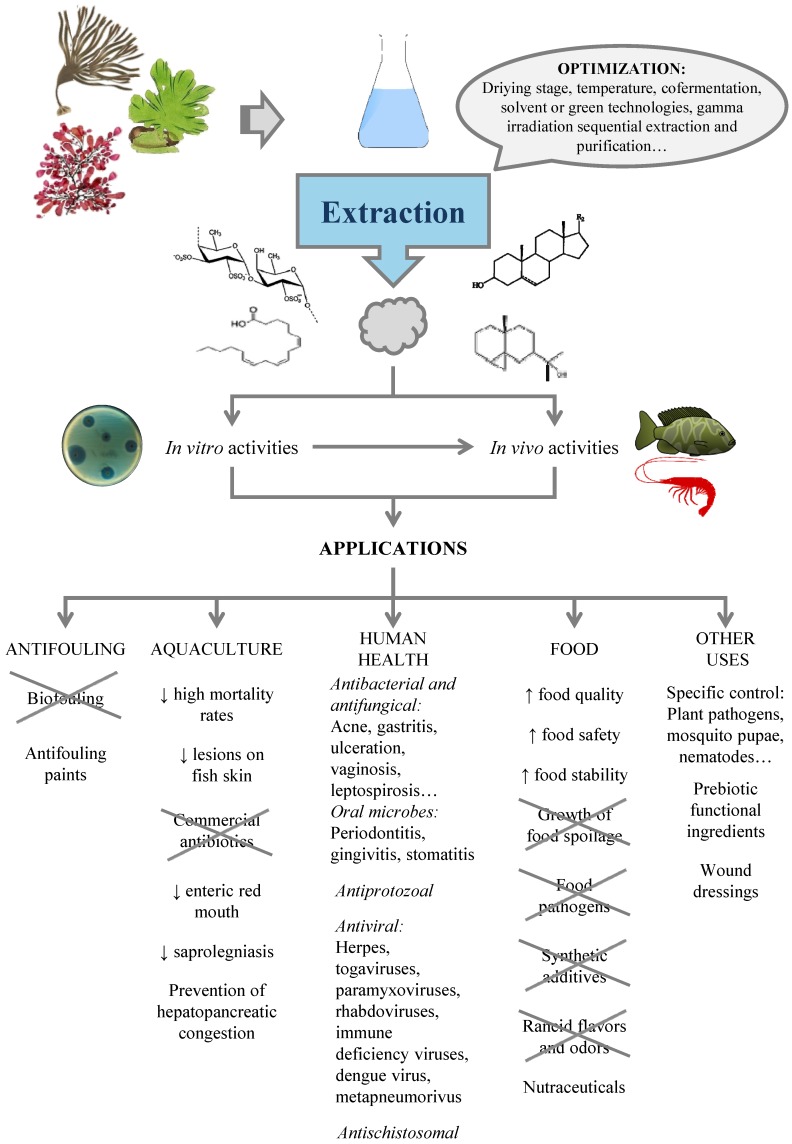
Stages proposed for the study and application of antimicrobials from seaweed, crossed out are the problems minimized or avoided.

A scheme of the different stages considered in this review and proposed for the utilization of antimicrobial compounds for a variety of applications is presented in Figure 3. The advantages related to the reduction or elimination of some problems are also indicated.

## 6. Conclusions and Future Trends

Marine algae are one of the largest producers of biomass in the marine environment and represent a potential source of new diverse and unique compounds. Many of the substances obtained from seaweed, such as alginates, carrageenan and agar have been used for decades in traditional medicine, pharmacology and food. Other compounds have bacteriostatic or antibacterial, antiviral, antitumor, anti-inflammatory and antifouling activities. Therefore, seaweed could provide promising bioactives that can be used in the treatment of human diseases, or new antimicrobial agents to replace synthetic antibacterial agents used in agriculture and in the food industry.

Much attention has been paid to the development of innovative projects for the pharmaceutical applications of seaweed, particularly in the design of novel antimicrobial drugs. Further investigations for the identification of promising algal species, standardization of analytical methods, isolation of compounds through bioassay guided fractionation, detailed chemical characterization and evaluation of their safety, as well as the evaluation of synergistic effects among the components, and efforts to enhance the yields and lower the extraction costs, are needed.

## Figures and Tables

**Table 1:** Antimicrobial activity of different solvent extracts from red seaweed.

Red Seaweed	Solvent	Organisms	Ref.
*Alsidium corallinum*	M	*Escherichia coli*, *Klebsiella pneumoniae*, *Staphylococcus aureus*	[49]
*Ceramium rubrum*	M	*E. coli*, *Enterococcus faecalis*, *S. aureus*	[49]
*Ceramium virgatum*	H/1:1 EE:Hp	*Salmonella enteritidis*, *E.coli*, *Listeria monocytogenes*, *Bacillus cereus*	[85]
*Chondrocanthus acicularis*	M	*E. coli*, *K. pneumoniae*, *E. faecalis*, *S. aureus*	[49]
*Chondracanthus canaliculatus*	E	*S. aureus*, *Streptococcus pyogenes*	[86]
*Chondrus crispus*	M	*L. monocytogenes*, *Salmonella abony*, *E. faecalis*, *P. aeruginosa*	[6]
*C. crispus*	95% E	*Pseudoalteromonas elyakovii*, *Vibrio aestuarianus*, *Polaribacter irgensii*, *Halomonas marina*, *Shewanella putrefaciens*	[75]
*C. crispus*	EA	*S. enteridis*, *E. coli*, *P. aeruginosa*, *L. innocua*, *B. cereus*, *S. aureus*, *L. brevis. E. faecalis*, *Candida sp.*	[74]
	DE	*E. coli*, *P. aeruginosa*, *L. innocua*, *B. cereus*, *S. aureus*, *L. brevis*, *E. faecalis*, *Candida sp.*	
	1:1 M:W	*E. coli*, *P. aeruginosa*, *L. innocua*, *B. cereus*, *S. aureus*, *L. brevis.* *E. faecalis*, *Candida sp.*	
*C. crispus*	95% E/T	*Cobetia marina, Marinobacter hydrocarbonoclasticus*	[87]
*Corallina elongata*	E, A, M	*B. subtilis*, *S. aureus, E. coli*, *Salmonella typhi*, *K. pneumoniae*, *Candida albicans*	[82]
*Corallina vancouverensis*	E	*S. aureus*, *S. pyogenes*	[86]
*Falkenbergia-phase of A. taxiformis*	M	*B. subtilis*, *K. pneumonia*, *P. aeruginosa, S aureus*, *S. epidermidis*, *Vibrio harveyi*, *V. alginolyticus*, *V. vulnificus*, *V. parahaemolyticus*, *V. alcaligenes*	[88]
*Ganonema farinosum*	E	*S. aureus*, *S. pyogenes*	[86]
*Gelidium attenatum*	M	*E. coli*, *K. pneumoniae*, *E. faecalis*, *S. aureus*	[49]
*Gelidium micropterum*	M	*V. parahaemolyticus*, *V. alcaligenes*	[88]
*Gelidium pulchellum*	M	*E. coli*, *E. faecalis*, *S. aureus*	[49]
*Gelidium pusillum*	M	*E. coli*, *K. pneumonia*, *E. faecalis*, *S. aureus*	[49]
	M	*V. harveyi*, *V. alginolyticus*, *V. vulnificus*, *V. parahaemolyticus*, *V. alcaligenes*	[88]
*Gelidium robustum*	E	*S. aureus*, *S. pyogenes*	[86]
*Gelidium spinulosum*	M	*E. coli*, *E. faecalis*, *S. aureus*	[49]
*Gracilaria dura*	2:1 C:M	*V. ordalii*, *V. alginolyticus*	[45]
*Gracilaria gracilis*	2:1 C:M	*V. salmonicida*	[45]
*Grateloupia livida*	E/PE	*S. aureus*, *E. coli*, *P. aeruginosa*	[89]
*Gracilaria ornata*	W	*E. coli*	[90]
*Gracilaria subsecundata*	E	*S. aureus*, *S. pyogenes*	[86]
*Gracilaria vermiculophylla*	EA	*S. enteridis*, *E. coli*, *P. aeruginosa*, *L. innocua*, *B. cereus*, *S. aureus*, *L. brevis. E. faecalis*	[74]
	DE	*S. enteridis*, *E. coli*, *P. aeruginosa*, *L. innocua*, *B. cereus*, *S. aureus*, *L. brevis. E. faecalis*, *Candida sp.*	
	1:1 M:W	*B. cereus*, *S. aureus*	
*Gracilariopsis longissima*	2:1 C:M	*V. alginolyticus*, *V. vulnificus*, *V. ordalii*, *V. salmonicida*	[45]
	M/2:1 C:M	*V. alginolyticus*, *V. fluvialis*, *V. vulnificus*, *V. cholerae non O-1*	[91]
*Grateloupia filicina*	M	*V. harveyi*, *V. alginolyticus*, *V. vulnificus*, *V. parahaemolyticus*, *V. alcaligenes*	[88]
*G. filicina*	M	*S.aureus*, *B. subtilis*, *E. coli*, *P. aeruginosa*	[83]
*Halopitys incurvus*	M	*E. coli*, *K. pneumoniae*, *E. faecalis*, *S. aureus*	[49]
*Hypnea musciformis*	M	*C. albicans*	[92]
*H. musciformis*	M	*K. pneumonia*, *E. faecalis*, *S. aureus*	[93]
	M	*E. coli*, *K. pneumoniae*, *E. faecalis*, *S. aureus*	[49]
*Hypnea pannosa*	M	*S. aureus*, *B. subtilis*, *E. coli*, *P. aeruginosa*	[83]
*Hypnea valentiae*	E	*S. aureus*, *S. pyogenes*	[86]
	M	*S. aureus*, *B. subtilis*, *E. coli*, *P. aeruginosa*	[83]
*Jania* *rubens*	E, A, M	*B. subtilis*, *S. aureus*, *E. coli*, *S. typhi*, *K. pneumoniae*, *C. albicans*	[82]
*Laurencia dendroidea*	A	*S. aureus*, *E. faecalis*	[94]
*Laurencia jonhstonii*	E	*S. aureus*, *S. pyogenes*	[86]
*Laurencia pacifica*	E	*S. aureus*, *S. pyogenes*	[86]
*Laurencia papillosa*	M	*S. aureus*, *B. subtilis*, *E. coli*, *P. aeruginosa*	[83]
	M/SC	*E. coli*, *P. aeruginosa*, *K. pneumoniae*, *Shigella flexineri*	
*Laurencia sp.*	M	*P. aeruginosa*, *S.epidermidis*, *V. harveyi*, *V. alginolyticus*, *V. vulnificus*, *V. parahaemolyticus*	[88]
*Neorhodomela larix*	E	*S. aureus*, *S. pyogenes*	[86]
*Ochtodes secundiramea*	A	*S. aureus*	[94]
*Osmundaria obtusiloba*	2:1 DCM:M	*P. aeruginosa*	[94]
*Plocamium cartilagineum*	M	*E. coli*, *E. faecalis*, *S. aureus*	[49]
*Polysiphonia lanosa*	W	*MRSA*,*S. aureus*, *E. cloacae*, *Clostridium perfringens*	[95]
*Polysphonia tuticorinensis*	M	*S. aureus*, *B. subtilis*, *E. coli*, *P. aeruginosa*	[83]
*Porphyra dioica*	EA	*E. coli*, *Bacillus cereus*, *Lactobacillus brevis.* *E. faecalis*, *Candida sp.*	[74]
	DE	*E. coli*, *B. cereus*, *L. brevis. E. faecalis*, *Candida sp.*	
	1:1 M:W	*S. aureus*, *E. faecalis*	
*Portieria horemanii*	M	*V. harveyi*, *V. alginolyticus*, *V. vulnificus*,	[88]
*Pterocladia capillacea*	E, A, M	*B. subtilis*, *S. aureus*, *E. coli*, *S. typhi*, *K. pneumoniae*, *C. albicans*	[82]
*Pterosiphonia complanata*	M	*E. coli*, *E. faecalis*, *S. aureus*	[49]
*Rhodymenia californica*	E	*S. aureus*, *S. pyogenes*	[86]
*Sphaerococcus coronopifolius*	M	*S. aureus*	[96]
	DCM/SC	*S. aureus*, *E. coli*, *P. aeruginosa*, *C. albicans*	
*Spyridia filamentosa*	M	*S. aureus*, *E. coli*, *E. faecalis*	[92]

A: acetone; C: chloroform; DCM: dichloromethane; DE: diethylether; E: ethanol; EA: ethyl acetate; EE: ethyl ether; H: hexane; Hp: heptane; M: methanol; T: toluene; W: water; SC: silica chromatography.

**Table 2:** Antimicrobial activity of different solvent extracts from green seaweed.

Green Seaweed	Extract	Organisms	Ref.
*Boodlea composita*	M	*V. harveyi*, *V. alginolyticus*, *V. vulnificus*, *V. parahaemolyticus*, *V. alcaligenes*	[88]
*Bryopsis pennata*	M	*V. vulnificus*, *V. parahaemolyticus*	[88]
*Caulerpa lentillifera*	M/EA	*E. coli*, *Staphylococcus aureus*, *Streptococcus sp.*, *Salmonella sp.*	[97]
*Caulerpa parvula*	M	*V. vulnificus*, *V. alcaligenes*	[88]
*Caulerpa racemosa*	M; M/DE; M/W	*E. coli*, *S. aureus*, *Streptococcus sp.*, *Salmonella sp.*	[97]
*Chaetomorpha aerea*	W	*Bacilus subtilis*, *Micrococcus luteus*, *S. aureus*	[98]
*Chaetomorpha linum*	2:1 C:M	*V. ordalii*, *V. vulnificus*	[45]
*Cladophora albida*	M	*V. harveyi*, *V. alginolyticus*, *V. vulnificus*, *V. parahaemolyticus*, *V. alcaligenes*	[88]
*Cladophora glomerata*	M	*V. fischeri*, *V. vulnificus*, *V. anguillarum*, *V. parahaemolyticus*	[99]
*Cladophora rupestris*	M, EA, E 2:1 C:M 2:1 C:M	*E. coli*, *Pseudomonas aeruginosa*, *S. aureus*, *V. harveyii*, *V. parahaemolyticus*, *V. alginolyticus* *Enterococcus sp.*, *Streptococcus agalactiae*, *V. fluvialis; V. salmonicida; V. vulnificus*, *V. ordalii; V. cholera non-O1; V. metschnikovii* *V. ordalii*, *V. salmonicida*, *V. vulnificus*	[68] [65] [45]
*Cladophora sp.*	E	*S. aureus*, *Streptococcus pyogenes*	[86]
*Codium amplivesiculatum*	E	*S. aureus*, *S. pyogenes*	[86]
*Codium cuneatum*	E	*S. aureus*, *S. pyogenes*	[86]
*Codium simulans*	E	*S. aureus*, *S. pyogenes*	[86]
*Enteropmorpha compressa*	M	*K. pneumoniae*, *V. harveyi*, *V. alginolyticus*, *V. vulnificus*, *V. parahaemolyticus*, *V. alcaligenes*	[88]
*E. compressa*	E	*Gardnerella vaginalis*	[100]
*E. compressa*	E, A, M	*B. subtilis*, *S. aureus*, *E. coli*, *S. typhi*, *K. pneumoniae*, *C. albicans*	[82]
*Enteromorpha intestinalis*	A	*Bacillus mycoides*, *B. subtilis*, *E. coli. K. pneumonia*, *S. aureus*, *A. flavus*, *A. fumigatus.* *C. albicans*, *P. purpurescens*, *P. verrucosum*	[101]
*Enteromorpha linza*	E, A, M	*B. subtilis*, *S. aureus*, *E. coli*, *S. typhi*, *K. pneumoniae*, *C. albicans*	[82]
*E. linza*	4:1 M:W E W/DCM	*Prevotella intermedia*, *Porphyromonas gingivalis* *G. vaginalis* *E. coli*, *Salmonella. typhimurium*	[102] [100] [103]
*Laurencia johnstonii*	1:1 A:M–Er	*S. aureus*	[3]
*Ulva dactilifera*	E	*S. aureus*, *S. pyogenes*	[86]
*Ulva fasciata*	E, A, M	*B. subtilis*, *S. aureus, E. coli*, *S. typhi*, *K. pneumoniae*, *C. albicans*	[82]
*Ulva lactuca*	E, A, M	*B. subtilis*, *S. aureus*, *E. coli*, *S. typhi*, *K. pneumoniae*, *C. albicans*	[82]
*U. lactuca*	1:1 A:M–Er	*S. aureus*	[3]
*U. lactuca*	A	*B. mycoides*, *B. subtilis*, *E. coli. K. pneumoniae*, *S. aureus*, *A. flavus*, *A. fumigatus.* *C. albicans*, *P. purpurescens*, *P. verrucosum*	[101]
*Ulva pertusa*	E	*G. vaginalis*	[100]
*U. pertusa*	M	*G. vaginalis*	[67]
*Ulva prolifera*	2:1 C:M	*V. ordali*	[45]

A: acetone; C: chloroform; DCM: dichloromethane; DE: diethylether; E: ethanol; EA: ethyl acetate; Er: ether; M: methanol; T: toluene; W: water.

**Table 3:** Antimicrobial activity of different solvent extracts from brown seaweed.

Brown Seaweed	Solvent	Organisms	Ref.
*Chnoospora implexa*	E	*S. aureus*, *S. pyogenes*	[86]
*Cladophora rupestris*	M	*E. coli*, *S. aureus*, *P. aeruginosa*, *V. harveyii*, *V. parahaemolyticus*, *V. alginolyticus*	[68]
*C. rupestris*	E	*E. coli*, *S. aureus*, *P. aeruginosa*, *V. harveyii*, *V. parahaemolyticus*, *V. alginolyticus*	[68]
*C. rupestris*	EA	*E. coli*, *S. aureus*, *P. aeruginosa*, *V. harveyii*, *V. parahaemolyticus*	[68]
*Colpomenia sinuosa*	E E, A, M	*S. aureus*, *S. pyogenes* *B. subtilis*, *S. aureus*, *E. coli*, *S. typhi*, *K. pneumoniae*, *C. albicans*	[86] [82]
*Colpomenia tuberculata*	E	*S. aureus*, *Sreptococcus pyogenes*	[86]
*Cystoseira osmundacea*	E	*S. pyogenes*	[86]
*Cystoseira trinodis*	M	*S. aureus*, *B. subtilis*, *E. coli*, *P. aeruginosa*	[83]
*Dictyopteris delicatula*	E	*S. aureus*, *S. pyogenes*	[86]
*Dictyopteris undulata*	E	*S. aureus*, *S. pyogenes*	[86]
*Dictyota dichotoma*	M	*S. aureus*, *B. subtilis*, *E. coli*, *P. aeruginosa*	[83]
*Dictyota flabellata*	E	*S. aureus*, *S. pyogenes*	[86]
*Dictyota indica*	M	*S. aureus*, *B. subtilis*, *E. coli*, *P. aeruginosa*	[83]
*Dictyota sp.*	2:1 DCM:M	*S. aureus*, *Enterococcus faecalis*, *P. aeruginosa*	[94]
*Eisenia bicyclis*	M; M/H; M/DCM; M/B	*S. aureus*, *S. epidermidis*, *Propionibacterium acnes*	[104]
*E. bicyclis*	M/EA	*S. aureus*, *S. epidermidis*, *P. acnes*, *P. aeruginosa*	[104]
*Fucus evanescens*	EA	*Hemophilus influenzae*, *Legionella pneumophila*, *Propionibacterium acnes S. pyogenes*, *Clostridium difficile*, *methicillin-resistant S. aureus*	[105]
*Himanthalia elongata*	H, E, W (PLE)	*Aspergillus niger*, *C. albicans*, *E. coli*, *S. aureus*	[106]
*H. elongata*	W, M and mixtures	*L. monocytogenes*, *E. faecalis*, *P. aeruginosa*, *S. abony*	[107]
*H. elongata*	C,DE,H/ 60% M:W/1:1 W:EA	*L. monocytogenes*, *S. abony*	[54]
*Hydroclathrus clathratus*	E	*S. aureus*, *S. pyogenes*	[86]
*Laminaria japonica*	W	*E. coli*, *S. aureus*, *B. cereus*	[108]
*Lobophora variegata*	M	*V. alginolyticus*, *V. vulnificus*, *V. parahaemolyticus*, *B. subtilis* *B.cereus*, *Micrococcus luteus*, *S. typhimurium*, *Aeromonas hydrophila*, *E. coli*	[88] [109]
*Padina concrescens*	M E	*V. alginolyticus*, *V. vulnificus*, *V. parahaemolyticus*, *B. subtilis* *S. aureus*, *S. pyogenes*	[88] [86]
*Padina mexicana*	E	*S. aureus*, *S. pyogenes*	[86]
*Padina pavonica*	M	*E. coli*	[92]
*Padina sanctae crucis*	M, E	*E. coli*, *S. aureus*, *P. aeruginosa*, *Candida tropicalis*, *C. kruzei*	[110]
*Padina tetrastomatica*	M	*V. alginolyticus*, *V. vulnificus*, *V. parahaemolyticus*, *B. subtilis*	[88]
*Rosenvingea intrincata*	E	*S. aureus*, *S. pyogenes*	[86]
*Saccharina japonica*	scW + AcH	*E. coli*, *S. typhimurium*, *S. aureus*, *B. cereus*	[111]
*S. japonica*	H, E	*E. coli*, *L. monocytogenes*, *S. aureus*	[112]
*S. japonica* **	SC-CO_2_ + E, 1:1 A:E	*E. coli*, *L. monocytogenes*, *B. cereus*, *S. aureus*, *C. albicans*, *A. brasiliensis*	[112]
*Sargassum binderi*	M, A	*S. aureus*, *B. subtilis*	[81]
*S. binderi*	EA	*B. subtilis*	[81]
*Sargassum flavellum*	M, A, EA	*B. subtilis*	[81]
*Sargassum horneri*	SC-CO_2_ + E, 1:1 A:E	*E. coli*, *L. monocytogenes*, *B. cereus*, *S. aureus*, *C. albicans*, *A. brasiliensis*	[112]
*S. horneri*	H, E	*E. coli*, *L. monocytogenes*, *S. aureus*	[112]
*Sargassum horridum*	E	*S. aureus*, *S. pyogenes*	[86]
*Sargassum myriocystum*	M	*S. aureus*, *B. subtilis*, *E. coli*, *P. aeruginosa*	[83]
*Sargassum plagyophillum*	M	*B.subtilis*, *S. aureus*	[81]
*Sargassum vulgare*	E, A, M	*B. subtilis*, *S. aureus*, *E. coli*, *S. typhi*, *K. pneumoniae*, *C. albicans*	[82]
*Scytosiphon lomentaria*	M	*S. aureus*, *S. typhimurium*, *E. coli*	[92]
*Stoe-chospermum marginatum*	M	*S. aureus*, *B. subtilis*, *E. coli*, *P. aeruginosa*	[83]
*Turbinaria ornata*	M	*S. aureus*, *B. subtilis*, *E. coli*, *P. aeruginosa*	[83]

AcH: acetic acid; A: acetone; B: butanol; C: chloroform; DCM: dichloromethane; DE: diethylether; E: ethanol; EA: ethyl acetate; H: hexane; M: methanol; scW: subcritical water; PLE: pressurized liquid extraction; W: water; Sequence os solvents; / fraction.
